# Strain induced anisotropy in liquid phase epitaxy grown nickel ferrite on magnesium gallate substrates

**DOI:** 10.1038/s41598-022-10814-8

**Published:** 2022-04-29

**Authors:** Ying Liu, Peng Zhou, Sudhir Regmi, Rao Bidthanapally, Maksym Popov, Jitao Zhang, Wei Zhang, Michael R. Page, Tianjin Zhang, Arunava Gupta, Gopalan Srinivasan

**Affiliations:** 1 Department of Physics, Oakland University, Rochester, MI 48309 USA; 2 Department of Materials Science and Engineering, Hubei University, Wuhan, 430062 China; 3 Center for Materials for Information Technology, The University of Alabama, Tuscaloosa, AL 3548 USA; 4 Faculty of Radiophysics, Electronics and Computer Systems, Taras Shevchenko National University of Kyiv, Kyiv, 01601 Ukraine; 5 College of Electrical and Information Engineering, Zhengzhou University of Light Industry, Zhengzhou, 450002 China; 6 Materials and Manufacturing Directorate, Air Force Research Laboratory, Wright-Patterson Air Force Base, Dayton, OH 45433 USA

**Keywords:** Materials science, Physics

## Abstract

This work focuses on the nature of magnetic anisotropy in 2.5–16 micron thick films of nickel ferrite (NFO) grown by liquid phase epitaxy (LPE). The technique, ideal for rapid growth of epitaxial oxide films, was utilized for films on (100) and (110) substrates of magnesium gallate (MGO). The motivation was to investigate the dependence of the growth induced anisotropy field on film thickness since submicron films of NFO were reported to show a very high anisotropy. The films grown at 850–875 C and subsequently annealed at 1000 C were found to be epitaxial, with the out-of-plane lattice constant showing unanticipated *decrease* with increasing film thickness and the estimated in-plane lattice constant increasing with the film thickness. The uniaxial anisotropy field *H*_*σ*_, estimated from X-ray diffraction data, ranged from 2.8–7.7 kOe with the films on (100) MGO having a higher *H*_*σ*_ value than for the films on (110) MGO. Ferromagnetic resonance (FMR) measurements for in-plane and out-of-plane static magnetic field were utilized to determine both the magnetocrystalline the anisotropy field *H*_*4*_ and the uniaxial anisotropy field *H*_*a*_. Values of *H*_*4*_ range from −0.24 to −0.86 kOe. The uniaxial anisotropy field *H*_*a*_ was an order of magnitude smaller than *H*_σ_ and it decreased with increasing film thickness for NFO films on (100) MGO, but *H*_*a*_ increased with film thickness for films on (110) MGO substrates. These observations indicate that the origin of the induced anisotropy could be attributed to several factors including (i) strain due to mismatch in the film-substrate lattice constants, (ii) possible variations in the bond lengths and bond angles in NFO during the growth process, and (iii) the strain arising from mismatch in the thermal expansion coefficients of the film and the substrate due to the high growth and annealing temperatures involved in the LPE technique. The LPE films of NFO on MGO substrates studied in this work are of interest for use in high frequency devices.

## Introduction

Ferrites and garnets are an important class of materials for studies on the nature of magnetism and for applications in a variety of technologies^1–5^. Spinel and hexagonal ferrites and yttrium iron garnet (YIG) and rare-earth iron garnets in general have ferrimagnetic ordering of magnetic moments with a large spontaneous magnetization at room temperature and a Curie temperature well above the room temperature. The magnetic ordering in these oxides with a large nonmagnetic substitution could be non-collinear, either canted or a spiral spin structure^6^. Several spinel and hexagonal ferrites and YIG have attracted interests in recent years for studies on their multiferroic properties, spin torque transfer phenomena and spintronics^7,8^. Ferrites and YIG have very high electrical resistivity and very low losses at high frequencies and are ideally suitable for use in microwave signal processing devices such as resonators, filters and phase shifters^9,10^.

Ferrite based microwave devices need a biasing magnetic field that could be provided by a permanent magnet^1^. Tuning the frequency of the devices, however, requires a source of variable magnetic field such as a solenoid or an electromagnet which would make the devices bulky, requiring a large power for operation, and cannot be miniaturized^1,9,10^. Yttrium iron garnet has one of the lowest losses at high frequencies amongst the ferrimagnetic oxides and is used in devices for 5–10 GHz range that may require a biasing field as high as 3 kOe^11,12^. Hexagonal ferrites with a large uniaxial or planar anisotropy fields on the order of 10–33 kOe are of interest for use in devices for the frequency range 20–110 GHz since the anisotropy field acts as a built-in biasing field and eliminates the need for very high biasing fields^13–15^. Recent reports on electric field tuning of ferrite devices through magneto-electric (ME) effects in composites with a ferroelectric^16–18^ or non-linear ME effects in hexagonal ferrites^19,20^ are significant for narrow band tuning and miniaturization of ferrite devices.

Although spinel ferrites such as nickel ferrite and lithium ferrite have low losses, there is lack of interests for their use in microwave devices due to the need for a large biasing field since they have a relatively small magneto-crystalline anisotropy field compared to a large uniaxial or easy plane anisotropy field, as high as ~ 10 to 33 kOe in hexagonal ferrites^21^. Single crystal nickel ferrite NiFe_2_O_4_ (NFO), for example, has a cubic magnetocrystalline anisotropy field of 0.5 kOe^21^. It is however possible to achieve a significant modification of magnetic parameters in thin films. A variety of techniques were used in the past to synthesize films of NFO, including electrodeposition^22^, chemical vapor deposition^23^, microwave-assisted solvothermal process^24^, pulsed laser deposition (PLD) ^25–29^, and liquid phase epitaxy (LPE)^30,31^. The objective in most of the past studies was to take advantage of strain due to film-substrate lattice mismatch to achieve a large growth induced anisotropy field. Recent reports of significance in this regard are (i) Zn and Al substituted nickel ferrite film on MgAl_2_O_4_ (MAO) substrate^26^ and (ii) nickel ferrite, NiFe_2_O_4_ (NFO) on MgGa_2_O_4_ (MGO), CoGa_2_O_4_ (CGO), and ZnGa_2_O_4_ (ZGO)substrates^27–29^. In the case of 15–57 nm thick films of Al and Zn substituted NFO on MAO substrates, low losses characterized by ferromagnetic resonance (FMR) line-widths of 5–40 Oe for the frequency range 5–35 GHz and a strain induced anisotropy field on the order of 10 kOe were reported^26^. A subset of current authors investigated the magnetic characteristics of 450 nm–1 ${\upmu }$m thick NFO films prepared by pulsed laser deposition on MGO, CGO and ZGO substrates with lattice mismatch of 0.8%, 0.2%, and 0.04%,respectively^27–29^. Growth induced in-plane anisotropy fields as high as 11.9 kOe for films on MGO, 0.5 kOe for CGO, and 0.1 kOe for ZGO were measured. The FMR line-widths increased from 25 Oe at 4 GHz to 75 Oe at 65 GHz^27^.

Although the very high anisotropy fields in some of these ultrathin films of NFO on MGO is of interest for use in high frequency devices, one requires films that are at least several microns thick for practical microwave devices. Techniques such as PLD or chemical vapor deposition techniques are therefore not appropriate for deposition of films with thickness suitable for microwave device applications. Liquid phase epitaxy (LPE) with growth rates as high as 1 ${\upmu }$m/(min) is ideally suitable for the deposition of thick ferrimagnetic spinel and garnet films^31–34^. We report here on the synthesis by LPE and structural and magnetic characterization of films 2.5–16 ${\upmu }$m NFO films on MGO substrates. The films were grown using a PbO-based flux at 850–875 C and annealed at 1000 C. Structural characterization by X-ray diffraction and electron microscopy and scanning probe microscopy (SPM) revealed epitaxial, single phase, stoichiometric NFO films of thickness 2.5–16 ${\upmu }$m on (100) MGO and 5–10 ${\upmu }$m on (110) MGO.

Estimates of the out-of-plane and in-plane lattice constants, *c* and *a*, respectively indicated a compressive strain perpendicular to the film plane and a tensile strain in-plane. The variation in *c* and *a* with film thickness revealed an *increase* in the magnitude of both strains with increasing film thickness. The strain induced anisotropy from XRD data predicted a uniaxial anisotropy *H*_σ_ ranging from 2.8 to 7.7 kOe, depending on the film thickness and substrate orientation. Ferromagnetic resonance (FMR) measurements were carried out to determine the magneto-crystalline anisotropy field *H*_*4*_ and growth induced anisotropy fields *H*_*a*_ in the films. These data indicate a switch from easy plane anisotropy field in the PLD films of thickness less than a micron^27–29^ to a uniaxial anisotropy in the thicker LPE films. For films on (100) MGO substrates, *H*_*a*_ decreased with increase in film thickness whereas an increase in *H*_*a*_ with increasing thickness was measured for films on (110) MGO. From the results of this study one has to conclude that in addition to strain due to the mismatch in NFO-MGO lattice constants, the strain produced by the mismatch in the thermal expansion coefficients of the film and the substrate, and possible changes in the bond lengths and bond angles in the NFO films could also be contributing factors to the induced anisotropy of NFO films..

## Experimental

**Figure 1:**
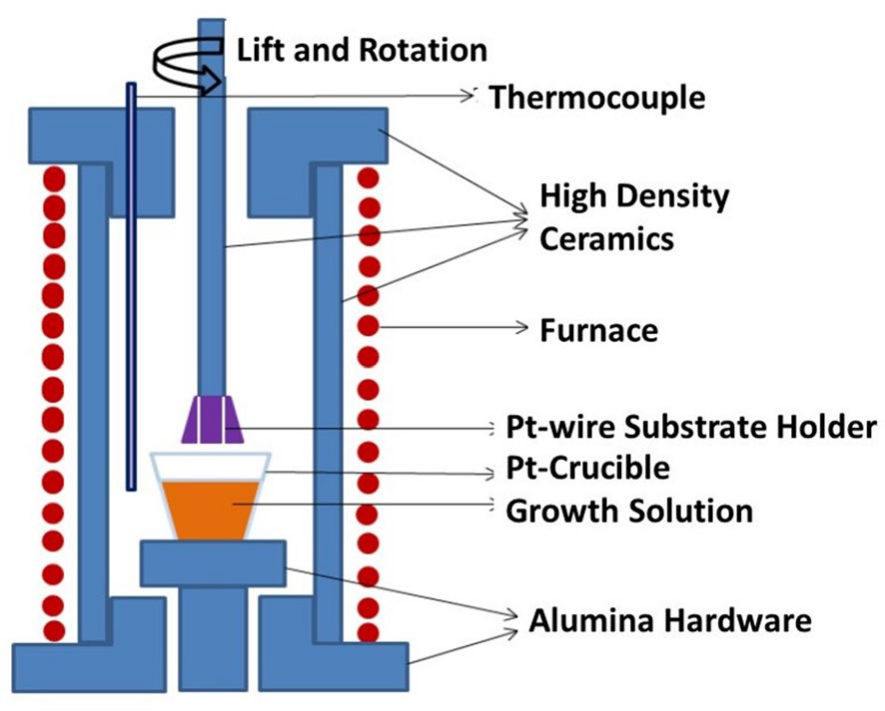
Schematic diagram of liquid phase epitaxy (LPE) crystal growth system.

Epitaxial Nickel ferrite (NFO) thin films were grown by LPE techniques^30–34^. Films were grown on 5 × 5 mm^2^, one side polished, (100) and (110) MgGa_2_O_4_ substrates. The lattice constants for the bulk NFO and MGO are 8.345 Å and 8.280 Å^27^, respectively, and the small film-substrate lattice mismatch of −0.78% is suitable for LPE growth. We have employed the standard LPE technique to grow the films under isothermal conditions from super-cooled melts consisting of PbO-B_2_O_3_ flux and NiO and Fe_2_O_3_ for the ferrite components. A vertical furnace with a constant temperature zone of 6 cm was used for this purpose. The schematics of our LPE system are shown in Fig. 1.

The growth method employed for the NFO films is similar to the procedure used for nickel zinc ferrite films reported previously in Ref.31. A melt consisting of 85.1PbO:8.75 B_2_O_3_:8.5 Fe_2_O_3_:0.85NiO mole percentages were used for the NFO film growth. The melt was homogenized at 1050 °C for 8–10 h and then cooled slowly to the growth temperature, T_g_ ~ 850–875 °C at a rate of 1 °C/min. Substrates held on platinum wires, in the vertical plane, were dipped into the melt at the growth temperature, under isothermal growth conditions, for a predetermined period and then raised from the melt slowly. The sample was allowed to cool and the excess flux adhering to the films was removed by cleaning in warm 20% acetic acid. The growth time varied from 5 to 30 min. The thickness of NFO films is dependent on the growth temperature and time and the substrate orientation. Following the growth, prior to structural and magnetic measurements, all the films were annealed in air at 1000 °C.

The structural characterization was performed by X-ray diffractometer (XRD) with a Cu K_α_ target (λ = 1.5406 Å), and the out-of-plane lattice constant of NFO film was obtained from the XRD data. The surface roughness and thickness of the film were measured using atomic force microscopy (AFM) and scanning electron microscopy (SEM), respectively. For magnetic characterization films on the MGO substrates were cut into 1 × 3 mm^2^ pieces after polishing off the films on the rough side. Magnetization measurements were done by vibrating sample magnetometry (VSM) using a Quantum Design system. Ferromagnetic resonance (FMR) measurements were done with the sample placed in an S-shaped coplanar waveguide and with the applied magnetic field either parallel or perpendicular to the sample plane.

## Results

**Figure 2:**
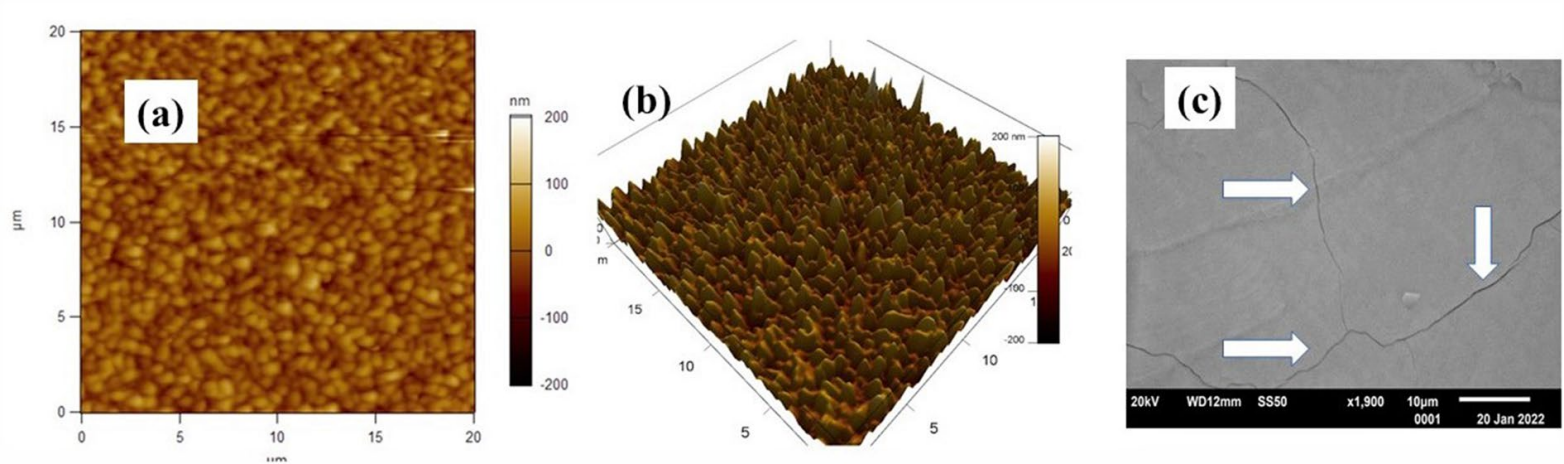
(**a**) 2D and (**b**) 3D AFM topography, and (**c**) SEM images of NFO film of thickness of 2.5 μm on (100) MGO substrate. The arrows indicate a few fine nanometer wide cracks on the film surface.

Nickel ferrite films on (100) and (110) MGO substrates were grown for 5 min to 30 min duration. Film growth rate varied from a minimum of 0.5 ${\upmu }$m/min to a maximum of 1.25 ${\upmu }$m/min. The growth time and the corresponding average film thickness measured by imaging the cross section with an SEM (Figs. S1–S4 in the Supplement), are listed in Table 1. The thickness varied from 2.5 ${\upmu }$m for a growth time of 5 min to a maximum of 16 ${\upmu }$m for a film grown for 30 min. A deviation in the thickness by ± 10% was inferred from SEM measurements and is expected in LPE film growth by vertical dipping. This procedure involves withdrawal of the film from the melt, after completion of the growth, over a period of several minutes. The film topography measured with an AFM showed them to have a smooth surface. Representative AFM topography image for a 2.5 ${\upmu }$m thick film on (100) MGO in Fig. 2 indicate a very smooth surface with a root mean square roughness of ~ 27 nm. Similar surface roughness was measured for other films (shown in Fig. S5 in the Supplement). Figure 2 also shows an SEM image of the film surface with a few nm wide surface cracks that could be due mismatch in the thermal expansion coefficients of NFO and MGO as discussed later in this section. The ferrite film chemical composition, measured by energy dispersive X-diffraction, was Ni_1.0±0.02_ Fe_2.0±0.04_ O_4_ (Figs. S1, S3 and S4 in the Supplement).

**Table 1:** Growth time and thickness of LPE grown NFO films on (100) and (110) MGO substrates.

Sample/film	Growth time	Thickness (μm)
NFO/(100) MGO	5 min	2.5
	15 min	10
	30 min	16
NFO/(110) MGO	5 min	5
	8 min	7.5
	15 min	10

**Figure 3:**
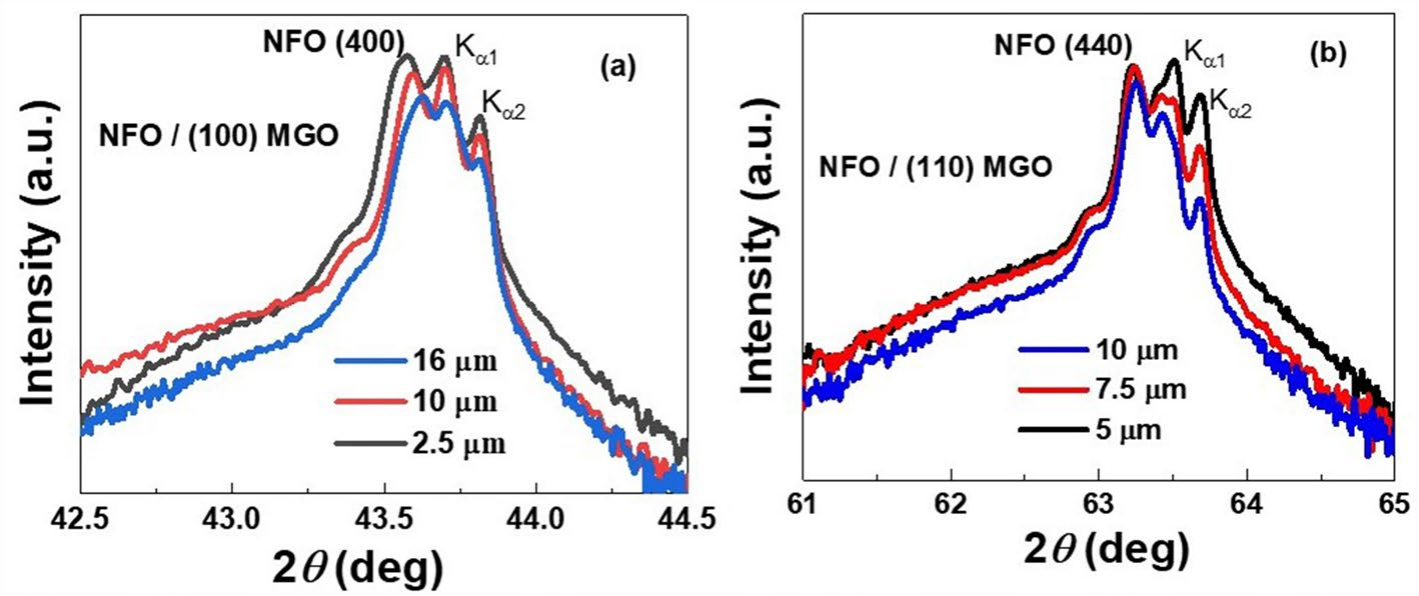
XRD pattern of (**a**) NFO films with the thickness of 2.5, 10, and 16 μm on (100) MGO, and (**b**) NFO films with thickness of 5, 7.5, and 10 μm on (110) MGO. The split peaks of substrates are from the K_α1_ and K_α2_ of Cu radiation wavelength.

The crystalline structure of the LPE films was analyzed by X-ray diffraction measurements. Figure 3 shows the XRD data of *θ*–2*θ* scans for annealed NFO films of thickness 2.5, 10 and 16 μm on (100) MGO and 5, 7.5 and 10 μm thick films on (110) MGO substrates. The films structures are spinels showing either (*h,0,0*) or (*h, k,0*) planes parallel to the film surface. Only the (*4,0,0*) and (*4,4,0*) diffraction peaks of NFO films on (100) and (110) MGO substrates, respectively, are shown in the figure and are indicative of the epitaxial nature of the films. The NFO peaks are close to the substrate peaks K_α1_ and K_α2_ of MGO and confirm good crystal growth along the *c*-axis.

The peaks of NFO film shift to the right toward the peaks of the substrate, i.e., to a higher angle with increase in film thickness, i.e., a *decrease in* the out of plane lattice constant *c* with increasing film thickness. The *c*-values estimated from data in Fig. 3 are listed in Table 2. The in-plane lattice constants *a* for the NFO films can be estimated from the unit cell volume for bulk NFO and *c-*values in Table 2 and are given by $a = \frac{{\sqrt {8.345^{3} } }}{c}$ Å. Values of *a* are also listed in Table 2.

**Table 2:** Out of plane lattice constant c determined from XRD data, estimated in-plane lattice constant a, and the strain induced anisotropy field H_σ_.

Sample	Thickness (μm)	Lattice constant *c* (Å)	Lattice constant *a* (Å)	*H*_σ_ (kOe)
NFO/(100) MGO	2.5	8.301	8.367	−6.4
	10	8.297	8.369	−7.0
	16	8.292	8.372	−7.7
NFO/(110) MGO	5	8.313	8.361	−2.8
	7.5	8.310	8.363	−2.9
	10	8.309	8.364	−3.0

The *c* values for all the six films in Table 2 are smaller than the lattice constant for bulk NFO and it decreases with increasing film thickness for films on both (100) and (110) MGO substrates. Similarly, the *a*-values for the films are higher than for bulk NFO and it increases with increasing film thickness. The most significant inference from the *c* and *a* value in Table 2 is the increase in both the out-of-plane compressive strain and in-plane tensile strain with increasing films thickness. These variations in *a* and *c* are contrary to the anticipated decrease in both strains with increasing film thickness due to relaxation of any growth induced strain due to the mismatch in the lattice constants of film and substrate.

The variations in the lattice constants with the film thickness in Table 2 is similar to the findings reported in Ref. 35 for films of yttrium iron garnet (YIG) on (100) yttrium aluminum garnet (YAG) with a lattice mismatch of −3%. In that study YIG films were deposited on YAG substrates by PLD techniques and then annealed at 800 C to obtain crystalline films. As in the present study, a similar increase in the in-plane tensile strain with increasing film thickness was attributed to the difference in the linear thermal expansion coefficient $\alpha$ for the film and the substrate. For YIG $\alpha$ = 10 × 10^–6^/C which is a factor 4 greater than that of YAG, $\alpha$ = 2.7 × 10^–6^/C^35,36^. In another study, the in-plane *tensile strain* in a 4 ${\upmu }$m thick YIG film on 1 mm thick YAG substrate grown by CVD techniques at 1250 C was also accounted for by the mismatch in $\alpha$^37^. In this study NFO films were grown on MGO at ~ 850C and annealed at 1000 C. The thermal expansion coefficient $\alpha$ = 13.4 × 10^–6^/C for NFO is 40% higher than $\alpha$ = 9.5 × 10^–6^/C for MGO^27,38,39^. The mismatch in $\alpha$-values, therefore, appears to be the cause of an unexpected increase in both the in-plane tensile strain and out-of-plane compressive strain as the film thickness and its volume are increased. The surface cracks seen in the SEM image of NFO film in Fig. 2c provide additional evidence for strain at the film-substrate interface due to mismatch in $\alpha$ values.

We estimated the strain induced magnetic anisotropy field *H*_σ_ from the lattice constants in Table 2 determined from the XRD data. The anisotropy field is given by^35,36^

1 $$H_{\sigma } = \, (3{\lambda}/M_{s} )\;\sigma$$

where λ is the magnetostriction, σ is the stress in the film and *M*_*s*_ is the saturation magnetization. The stress is given by^27^,

2 $$\sigma = E\left( {{2}a{-}c{-}a_{bulk} } \right)/a_{bulk}$$

where *E* is the Young’s modulus. Using the bulk single crystal values of *M*_*s*_ ≈ 278 emu/cc (*4* $\pi$*M*_*s*_ = 3.5 kG)^21^, magnetostriction *λ*_100_ =  − 46 ppm and *λ*_*110*_ =  − 26 ppm for bulk NFO^40,41^, the Young’s modulus *E* = 1.22 × 10^12^ dyne/cm^2^,^27^ *a*_*bulk*_ = 0.8345 nm, calculated values of $H_{\sigma }$ are given in Table 2. The estimates predict a uniaxial anisotropy field perpendicular to the film plane in all of the films, with the *H*_σ_ values higher for films on (100) MGO than for films grown on (110) MGO. An increase in the uniaxial anisotropy is expected with the increase in film thickness for films on both (100) and (110) MGO substrates.

**Figure 4:**
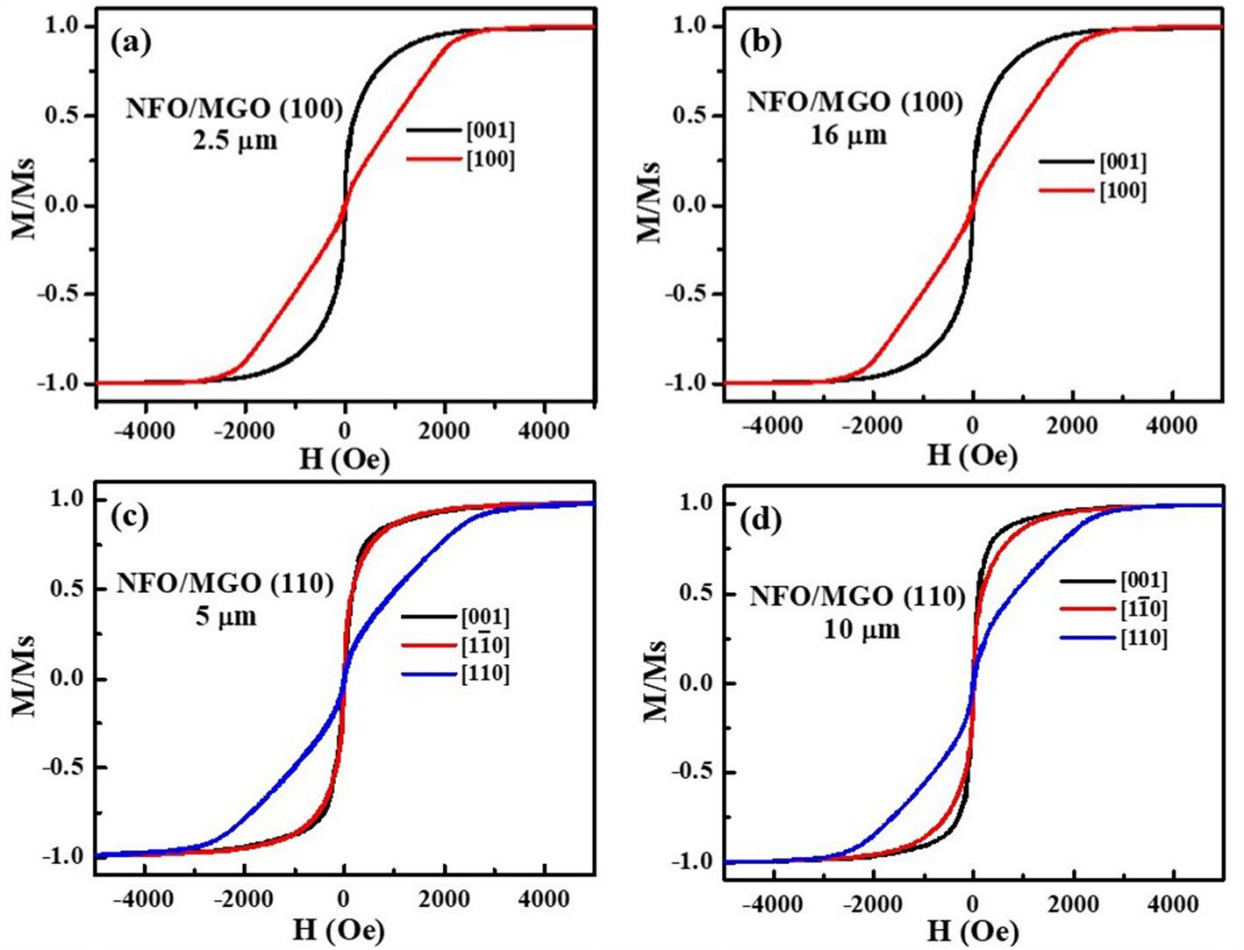
The normalized magnetization (M/M_s_) vs magnetic field H for NFO/MGO (100) with thicknesses of (**a**) 2.5 μm and (**b**) 16 μm for in-plane H along [001] direction and for out-of-plane H along [100]. Similar M/M_s_ vs H data for NFO/MGO (110) with thicknesses of (**c**) 5 μm and (**d**) 10 μm for in-plane H along [001] and [1$\overline{1}$0] directions and for out-of-plane H along [110] direction.

Magnetic characterization of the films by measurements of (i) magnetization and (ii) ferromagnetic resonance is considered next. The normalized magnetization *M/M*_*s*_ vs static magnetic field *H* for the NFO films grown on (100) and (110) MGO are shown in Fig. 4. The measurements were made at room temperature for *H* parallel and perpendicular to the sample plane. For NFO/(100) MGO, in-plane *H* was applied along[001] and out-of-plane *H* along [100]. For films of NFO on (110) MGO, magnetization data were obtained for in-plane *H* along [001] and [1${\overline{\text{1}}}$0] and out-of-plane *H* along [110]. Data for in-plane fields for different *H* orientations have saturation of *M* at approximately for the same *H* value. The magnetic hysteresis loops in Fig. 4 show saturation of *M* for in-plane *H* at a much lower value compared to *H* perpendicular to the sample plane for all the NFO films.

The saturation magnetization was estimated using both the film volume as well as the film mass and the x-ray density of 5.4 g/cc for NFO. The *M*_*s*_ value estimated using the film volume ranged from a minimum of 200 emu/cc to a maximum of 278 emu/cc whereas *4*$\pi$*M*_*s*_ calculated from the film mass ranged from 3.4 to 3.6 kG. As mentioned earlier, the film thickness data showed a deviation of ± 10%. Such a large uncertainty in the thickness is expected in the case of LPE growth by vertical dipping method. One, therefore, anticipates a similar uncertainty in the estimated *M*_*s*_ using the measured film thickness. Our current value of *4*$\pi$*M*_*s*_ = 3.5 ± 0.1 kG agrees with previously reported values for PLD films of NFO on MGO^28^.

The saturation field *H*_*s*_ for out of plane static field for all the NFO films that were obtained from the magnetic hysteresis loops in Fig. 4 are given in Table 3. The value of *H*_*s*_ can be written as^27^

3 $$H_{s} = H_{4} + \, 4\pi M_{s} + \, H_{\sigma } ,$$

where *H*_*4*_ is four-fold symmetric cubic magnetocrystalline anisotropy field (and its value determined from FMR are given in Table 3 as discussed next) and *4πM*_*s*_ = 3.5 kG*.* Thus *H*_σ_, the strain induced anisotropy field, can be determined from Eq. (3). The estimated *H*_σ_ value is given in Table 3. The anisotropy fields are an order of magnitude smaller than estimated *H*_σ_ from XRD data and do not show any systematic variation with film thickness or substrate orientation.

**Table 3:** Saturation magnetic field H_s_ for out-of-plane hysteresis loops, magneto-crystalline anisotropy determined from FMR (Table 4), and growth induced anisotropy field H_σ_ determined from magnetization versus magnetic field data for NFO films on (100) and (110) MGO substrates with different thickness.

Sample	Thickness (μm)	H_s_ (kOe)	H_*4*_ (kOe) (from FMR)	H_σ_ (kOe)
NFO/(100) MGO	2.5	2.5	−0.51	−0.49
	10	3.5	−0.50	0.50
	16	2.5	−0.24	−0.76
NFO/(110) MGO	5	3.2	−0.48	0.18
	7.5	2.5	−0.75	−0.75
	10	2.6	−0.86	−0.04

**Figure 5:**
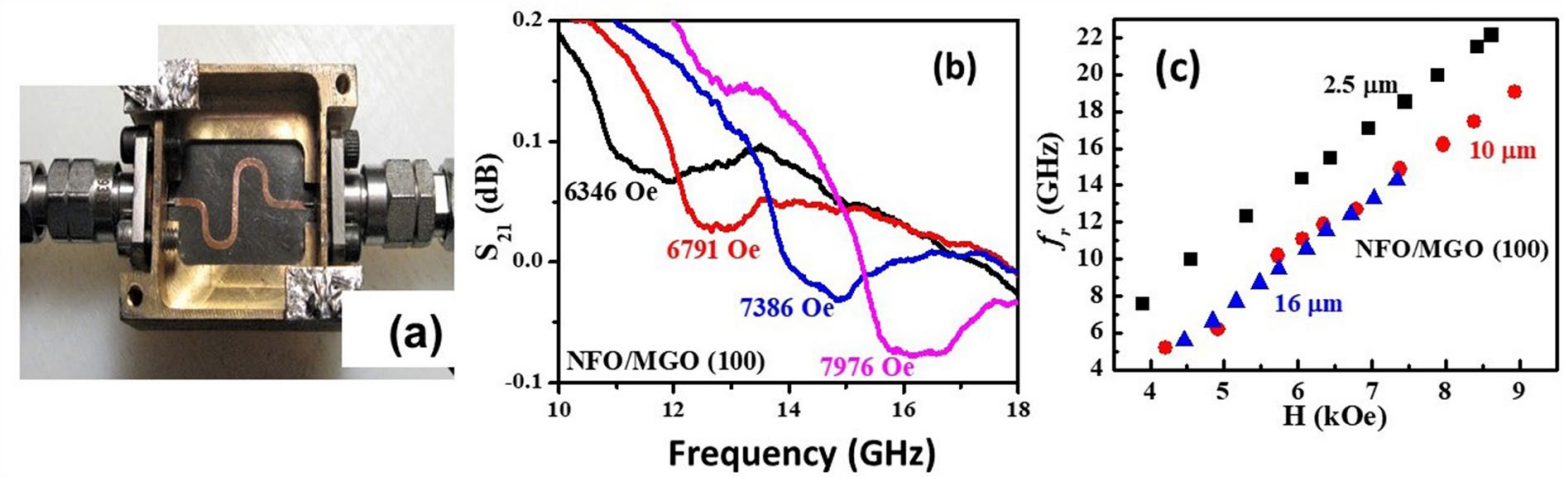
(**a**) Coplanar waveguide microwave excitation structure used for FMR measurements. (**b**) Profiles of S_21_ amplitude vs frequency for a series of magnetic fields perpendicular to the film plane for NFO film with thickness of 10 μm on (100) MGO. (**c**) Resonance frequency f_r_ obtained from profiles as a function of H for NFO films with thickness of 2.5, 10 and 16 μm on (100) MGO.

**Figure 6:**
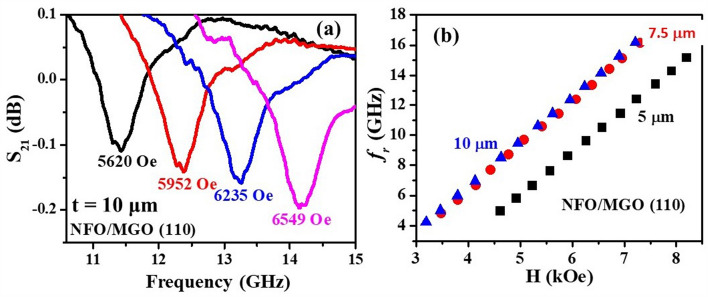
(**a**) Out of plane FMR data as in Fig. 5(b) for NFO film with thickness of 10 μm on (110) MGO and (**b**) f_r_ vs H data for NFO films with thickness of 5, 7.5 and 10 μm on (110) MGO substrates.

Ferromagnetic resonance measurements on the NFO films are considered next. Measurements were done with the sample placed in a coplanar waveguide and excited with microwave power from a vector network analyzer. Profiles of scattering matrix *S*_*21*_ vs *f* for series of *H* were recorded for *H* either perpendicular to the sample plane or in-plane *H* applied parallel to [001] or [011] directions. Figure 5a shows the coplanar waveguide used for the FMR measurements. Figure 5b shows representative profiles of the scattering parameter *S*_*21*_ vs *H* for the 10 μm thick NFO and the resonance appears as a dip in the power transmitted through the coplanar waveguide (additional profiles for NFO/(100) MGO are shown in Figs. 6 and 7 in the Supplement). Figure 5b shows the resonance frequency *f*_*r*_ vs *H* for all three NFO films on MGO. A linear increase in *f*_*r*_ with *H* is evident for all the films.

**Figure 7:**
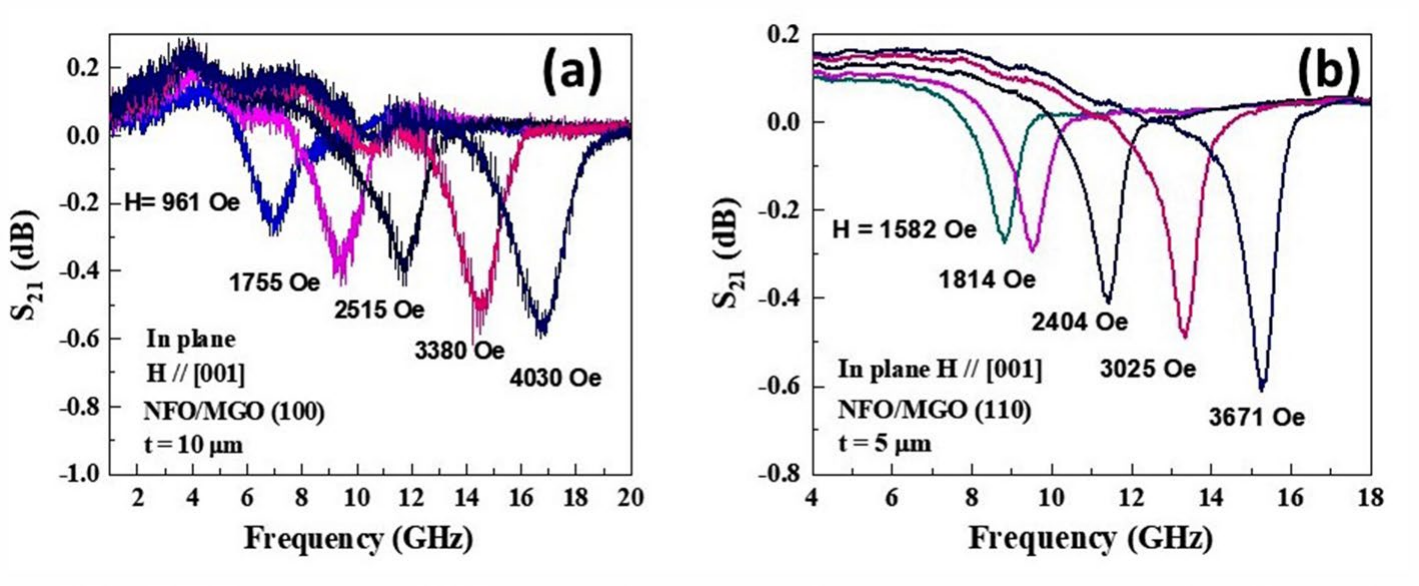
(**a**) S_21_ vs f profiles as a function of H applied parallel to [001] direction for 10 ${\upmu }$ m thick NFO on (100) MGO. (**b**) Profiles as in (**a**) for H//[001] for 5 ${\upmu }$m thick film of NFO on (110) MGO.

Similar FMR data for films on (110) MGO substrate are shown in Fig. 6. Figure 6a shows FMR spectra of NFO film with a thickness of 10 μm grown on (110) MGO substrate (additional profiles for NFO/(110) MGO shown in Figs. 8 and 9 in the Supplement). Figure 6(b) shows the resonance frequency *f*_*r*_ as a function of *H* for (110) NFO films with thickness of 5, 7.5 and 10 μm.

A linear increase in *f*_*r*_ with *H* is also seen for all the films. The resonance condition for out-of-plane FMR is given by^42^

4 $$f_{r} =\gamma \left( {H {-} 4\pi M_{eff}} \right)$$

where γ is the gyromagnetic ratio and the effective magnetization *4πM*_*eff*_ = *4πM*_*s*_ + *H*_*a*_ + *H*_*4*_. In Eq. (4) *H*_*a*_ is the growth induced uniaxial anisotropy field and it is positive for easy plane anisotropy and negative for uniaxial anisotropy field perpendicular to sample plane. We utilized linear fits to data on resonance frequency *f*_*r*_ vs *H* from profiles as in Figs. 5 and 6 to estimate the parameters γ and *4πM*_*eff*_ and are given in Table 4. The values of *H*_*a*_ and the magnetocrystalline anisotropy field *H*_*4*_ were determined from FMR data for in-plane *H* and *4πM*_*eff*_.in Table 4 as described next. FMR measurements for in-plane *H* were done for (i) *H* parallel to [001] and [011] directions for NFO on (100) MGO and (ii) for *H* parallel to [001] for NFO films on (110) MGO. Figure 7 shows *S*_*21*_ vs *f* for a series of in-plane *H*-values for the 10 ${\upmu }$m thick NFO on (100) MGO.

**Table 4:** The effective magnetization 4 $\pi$ M_eff_, gyromagnetic ratio γ and the calculated uniaxial anisotropy field H_a_ and magnetocrystalline anisotropy field H_4_ determined from FMR profiles of NFO films on (100) and (110) MGO substrates.

Sample	Thickness (μm)	4 $\pi$ M_eff_ (kOe)	γ (GHz/kOe)	H_a_ (kOe)	H_4_ (kOe)
NFO/(100) MGO	2.5	1.3	3.03	−1.7	−0.51
	10	2.5	2.99	−0.5	−0.50
	16	2.65	3.05	−0.3	−0.24
NFO/(110) MGO	5	2.88	2.84	−0.1	−0.48
	7.5	1.85	2.96	−1.1	−0.75
	10	1.79	2.98	−1.2	−0.86

The resonance conditions for in-plane *H* along [001] and [011] are given by^27^:

5 $$H// \, \left[ {00{1}} \right]{:}\; f_{r} = \gamma \left( {H - H_{4} } \right)^{1/2}\; (H \, + \, H_{4} /2 + \, 4\pi M_{s} + \, H_{a} )^{1/2}$$

6 $${\text{and}} \quad H {} \left[ {011} \right]{:}\; fr = \gamma\left( {H + H_{4} }\right)^{1/2}\; \left( {H + H_{4} + 4\pi Ms + Ha} \right)^{1/2}.$$

Equations (5) and (6), data on in-plane FMR resonance frequencies vs *H*, and values of γ and *4*$\pi$*M*_*eff*_ in Table 4 and 4$\pi$*M*_*s*_ = 3.5 kG were used in the estimates of the values of *H*_*a*_ and *H*_*4*_ that are listed in Table 4. The γ values are in the range 2.84–3.03 GHz/kOe for all the NFO films, in agreement with the value of 3.0 for single crystal NFO^21,27^. The magnetocrystalline anisotropy field *H*_*4*_ ranges from −0.24 kOe to −0.51 kOe for films on (100) MGO and is higher in films on (110) MGO with values—0.48 kOe to −0.86 kOe. Most of the *H*_*4*_ values are higher than −0.5 kOe for bulk single crystal NFO^21^. The anisotropy field *H*_*a*_ is negative for films on both kinds of MGO substrates, indicating that *H*_*a*_ is uniaxial in nature and perpendicular to the film plane. Films on (100) MGO show a decrease in *H*_*a*_ with increase in the thickness of NFO, whereas films on (110) show an increase in *H*_*a*_ with film thickness.

## Discussion

The primary objective of this work was to determine the magnetic parameters, the growth induced anisotropy fields, in particular, in NFO films grown by LPE techniques on MGO substrates. This was accomplished by determining *H*_σ_ from XRD and *M* vs *H*, and *H*_*a*_ from FMR measurements. The anisotropy fields for the LPE films are listed in Table 5 and, for comparison, the parameters for submicron films of NFO prepared by PLD on MGO substrates are also listed. First, we consider results of the XRD measurements. The out-of-plane and in-plane lattice constants, *c* and *a*, respectively calculated from XRD data in Fig. 3 revealed a compressive strain perpendicular to the film plane and a tensile strain in-plane and both increased with increasing film thickness for films on both (100) and (110) MGO substrates and resulting in a uniaxial growth induced anisotropy field *H*_σ_ along the *c*-direction. Thus, with increasing film thickness there is no evidence from XRD data for the expected relaxation of distortion due to the mismatch in the film-substrate lattice constants. As discussed in the previous section, the variations in *c* and *a* with the film thickness in both kinds of NFO films could possibly be due to the difference in their thermal expansion coefficients for the film and the substrate since the LPE synthesis of the film involved high growth and annealing temperatures. Such a finding was previously reported in the case of PLD YIG films on YAG substrates^35,37^. Estimated *H*_σ_ values (Tables 2 and 4) are larger for films on (100) MGO than for films on (110) MGO and showed an increase with increase in the thickness of NFO. Thus, the thick LPE grown films are expected to show out-of-plane easy axis anisotropy whereas thinner 450 nm NFO film on (100) MGO was reported to have an easy plane type anisotropy *H*_σ_ = 13.4 kOe from XRD data^27^. Films of NFO with thickness 445–460 nm deposited by PLD on MgAl_2_O_4_ (MAO) and CoGa_2_O_4_ (CGO) substrates were also reported to have in-plane *H*_σ_ ranging from 2.8 kOe for the film on CGO to 14.2 kOe for the film on MAO^27^.

**Table 5:** Growth induced anisotropy field H_σ_ = H_a_ for LPE grown films of NFO on MGO substrates. Parameters for PLD grown submicron thick films of NFO on MGO are also listed for comparison.

Sample	Thickness (μm)	H_σ_ (kOe) (XRD)	H_σ_ (kOe) (M vs H)	H_a_ (kOe) (FMR)
NFO/(100) MGO	0.45	13.4^27^		11.9^27^
				8.2^28^
	0.6			10.4^28^
	2.5	−6.4	−0.49	−1.7
	10	−7.0	0.50	−0.5
	16	−7.7	−0.76	−0.6
NFO/(110) MGO	0.6	–	–	4.75^28^
	5	−2.8	0.18	−0.1
	7.5	−2.9	−0.75	−0.9
	10	−3.0	−0.04	−0.85

Determination of *H*_σ_ from magnetic hysteresis loop data is considered next. Since the saturation field *H*_*s*_ was higher for out of plane loop than for in-plane loop, the anisotropy field was determined from *H*_*s*_ values. The *H*_σ_ values (Tables 3 and 5) are rather small, more than an order of magnitude smaller than values estimated from XRD data, and range from −0.76 kOe to 0.5 kOe. There is also no systematic variation in *H*_σ_ values with the film thickness for the films on both (100) and (110) MGO substrates. Although similar measurements on submicron thick films of NFO on MGO, CGO and MAO substrates yielded *H*_σ_ values comparable to estimates from XRD data^27^, one has to infer that the procedure is not sensitive enough in the case of thick LPE films.

Magnetic parameters obtained from data on the resonance frequency *f*_*r*_ vs *H* for in-plane and out-of-plane FMR yielded values of the magnetocrystalline anisotropy (MCA) field *H*_*4*_ and growth induced anisotropy field *H*_*a*_ (= *H*_σ_) listed in Tables 4 and 5. The MCA shows a decrease with film thickness for films on (100) MGO, but it increases with film thickness for films on (110) MGO. Most of the *H*_*4*_ values in Table 4 are higher than the single crystal value of −0.512 Oe^21^ that could be due to variations in the bond angle and bond lengths and a tetragonal distortion in the films. Thin films of NFO on MGO, ZGO, and on MgAl_2_O_4_ are also reported to have *H*_*4*_ = −78 Oe to −370 Oe that are smaller than the bulk single crystal values^27,29^. Nickel ferrite film prepared by laser molecular beam epitaxy (MBE) on (001) STO was reported to have *H*_*4*_ = −0.29 kOe^43^. Thus, the *H*_*4*_ for our LPE films are in the same range with reported values for thin films prepared by several different techniques.

Anisotropy fields *H*_*a*_ for thin PLD films of NFO on MGO reported by subset of current authors are also listed in Table 5 for comparison^27,28^. In the case of PLD NFO films on (100) MGO, *H*_*a*_ = 8.2–11.9 kOe is easy plane type for 450–600 nm films^27,28^. For our thicker LPE films on (100) MGO, however, *H*_*a*_ is uniaxial, decreases with increasing film thickness, an order of magnitude smaller than *H*_*a*_ for thin PLD films, and much smaller than *H*_σ_ in Table 3 estimated from XRD data. For films on (110) MGO, 600 nm thick PLD film is reported to show a large in-plane *H*_*a*_ whereas our thicker LPE films have a relatively small *H*_*a*_ that increases with increasing film thickness. Thus, the key inferences from *H*_*a*_ values for LPE films are (i) the anisotropy is uniaxial and much smaller than easy plane anistropy reported for thin PLD films of NFO and (ii) *H*_*a*_ decreases with films thickness for (100) MGO and shows an increase with film thickness for (110) MGO.

Finally, we compare *H*_*a*_ values determined by FMR in pure and substituted nickel ferrite films on a variety of substrates with the values for LPE films in Table 5. In 445–465 nm thick NFO films on ZGO, CGO and MAO, in-plane FMR measurements yielded easy plane type *H*_*a*_ values of 0.1 kOe, 0.5 kOe and 2.6 kOe, respectively. We recently carried out studies on 2–30 ${\upmu }$ m thick LPE grown Ni_0.85_Zn_0.15_Fe_2_O_4 (_NZFO) films on (111) and (100) MGO substrates^31^. An induced in-plane anisotropy field in the films was evident from the magnetization and FMR measurements. The anisotropy field *H*_*a*_ was in the range 1.2 to 1.4 kOe in the films on (111) MGO and *H*_*a*_ = 2.6 to 2.9 kOe for the films on (100) MGO. Similarly, very thin PLD films of NiZnAl-ferrite on MgAl_2_O_4_ were found to have an easy plane *H*_*a*_ of 10 kOe^26^. Films of NFO 25–50 nm in thickness made by laser MBE showed in-plane anisotropy field of 0.5 kOe^43^. Our recent report on uniaxial growth induced anisotropy in 50 nm films of NFO on SrTiO_3_ substrates is of relevance to this study^44^. Films of NFO were deposited by PLD techniques on (001), (110), and (111) SrTiO_3_ (STO) single-crystal substrates. The lattice mismatch between film and substrate was very high, on the order of 6.7%. A uniaxial anisotropy was evident from magnetization and FMR measurements and NFO films on (111) STO and (001) STO revealed uniaxial anisotropy *H*_*a*_ = 1.4–6.1 kOe. Thin NFO films on STO and thick LPE films of NFO on MGO are somewhat unique with uniaxial growth induced anisotropy field.

To summarize, the strain that contributes to the anisotropy field in our LPE films processed at high temperatures seems to originate from mismatch both in the lattice constants and thermal expansion coefficients of the film and substrate. The variations in the magnetocrystalline anisotropy field in the films also indicate growth induced changes in the bond lengths and bond angles that will contribute to changes in other magnetic order parameters. Films of NFO with a thickness of 2.5 ${\upmu }$m on (100) MGO has the highest value of *H*_*a*_ in Table 5 for the LPE films. Follow up studies on the effects of annealing temperature and deposition and characterization of LPE films with thickness 1–2 ${\upmu }$m are essential to come up with optimum annealing conditions and film thickness for enhanced *H*_*a*_*.*

## Conclusions

Our investigations on LPE grown nickel ferrite films on magnesium gallate substrates revealed a switch from easy plane anisotropy reported in submicron thick films to a uniaxial growth induced anisotropy. NFO films with thickness ranging from 2.5 ${\upmu }$m to 16 ${\upmu }$m were grown on (100) and (110) substrates of MGO with a film-lattice mismatch of 0.78%. Structural characterization by X-ray diffraction indicated a compressive out-of-plane strain with the lattice constant *c* that decreased with increase in the film thickness and smaller lattice constant than for bulk NFO. The in-plane lattice constant *a* increased with the film thickness. The perpendicular anisotropy field estimated from XRD data was in the range 2.8–7.7 kOe, increased with increasing films thickness, and was higher in films on (100) MGO than for films on (110) MGO. Ferromagnetic resonance measurements on the films provided clear evidence for a switch from a large easy plane anisotropy field reported in 450–600 nm thick NFO films on MGO to a smaller uniaxial anisotropy field in the thick LPE films. The cause of the growth induced anisotropy in our films is possibly due to mismatch in the lattice constants and thermal expansion coefficients of NFO and MGO and other contributing factors such as variation in bond angles and bond lengths. Values of *H*_*a*_ calculated from the data on resonance frequency vs *H* were in the range 0.3 to 1.7 kOe for films on (100) MGO and 0.1 to 1.2 kOe for films on (110) MGO. The thick NFO films on MGO substrates showing uniaxial anisotropy are of interest for memory devices and high frequency device applications.

## Supplementary Information

Supplementary Information.

## References

[1] Adam JD, Davis LE, Dionne GF, Schloemann EF, Stitzer SN (2002). Ferrite devices and materials. IEEE Trans. Microw. Theory Tech..

[2] Özgür Ü, Alivov Y, Morkoç H (2009). Microwave ferrites, part 1: fundamental properties. J. Mater. Sci.: Mater. Electron..

[3] Mallmann EJJ, Sombra ASB, Goes JC, Fechine PBA (2013). Yttrium iron garnet: properties and applications review. Solid State Phenom..

[4] Pullar RC (2012). Hexagonal ferrites: A review of the synthesis, properties and applications of hexaferrite ceramics. Progr. Mater. Sci..

[5] Narang SB, Pubby K (2021). Nickel spinel ferrites: A review. J. Magn. Magn. Mater..

[6] Utsumi S, Tanaka S, Maruyama K, Hatakeyama N, Itoh K, Koike J, Horikawa A, Iriyama H, Kanamaru H, Amako Y, Iiyama T (2020). Flux growth and magnetic properties of helimagnetic hexagonal ferrite Ba (Fe1–x Sc x) 12O19 single crystals. ACS Omega.

[7] Ramaswamy R, Lee JM, Cai K, Yang H (2018). Recent advances in spin-orbit torques: Moving towards device applications. Appl. Phys. Rev..

[8] Ebnabbasi K, Mohebbi M, Vittoria C (2013). Room temperature magnetoelectric effects in bulk poly-crystalline materials of M-and Z-type hexaferrites. J. Appl. Phys..

[9] Pardavi-Horvath M (2000). Microwave applications of soft ferrites. J. Magn. Magn. Mater..

[10] Schloemann E (2000). Advances in ferrite microwave materials and devices. J. Magn. Magn. Mater..

[11] Schmidt G, Hauser C, Trempler P, Paleschke M, Papaioannou ET (2020). Ultra thin films of yttrium iron garnet with very low damping: A review. Physica Status Solidi (b).

[12] Sharma V, Saha J, Patnaik S, Kuanr BK (2017). Synthesis and characterization of yttrium iron garnet (YIG) nanoparticles-Microwave material. AIP Adv..

[13] Glass HL (1988). Ferrite films for microwave and millimeter-wave devices. Proc. IEEE.

[14] Ustinov AB, Tatarenko AS, Srinivasan G, Balbashov AM (2009). Al substituted Ba-hexaferrite single-crystal films for millimeter-wave devices. J. Appl. Phys..

[15] Popov M, Zavislyak I, Ustinov A, Srinivasan G (2011). Sub-terahertz magnetic and dielectric excitations in hexagonal ferrites. IEEE Trans. Magn..

[16] Sun NX, Srinivasan G (2012). Voltage control of magnetism in multiferroic heterostructures and devices. SPIN.

[17] Liang X, Chen H, Sun NX (2021). Magnetoelectric materials and devices. APL Mater..

[18] Ramesh R, Martin LW (2021). Electric field control of magnetism: multiferroics and magnetoelectrics. Riv. Nuovo Cim..

[19] Popov MA, Zavislyak IV, Srinivasan G (2018). Current tunable barium hexaferrite millimeter wave resonator. Microw. Opt. Technol. Lett..

[20] Zavislyak IV, Popov MA, Srinivasan G (2016). Current-induced nonlinear magnetoelectric effects in strontium hexaferrite. Phys. Rev. B.

[21] Landolt-Bornstein; *Numerical data and functional relationships in science and technology, Group III, Crystal and Solid State Physics,* vol 4(b), *Magnetic and Other Properties of Oxides*, eds. K.-H. Hellwege and A. M. Springer, Springer-Verlag, New York (1970).

[22] Lokhande CD, Kulkarni SS, Mane RS, Joo OS, Han SH (2011). Magnetic studies on one-step chemically synthesized nickel ferrite thin films. Ceram. Int..

[23] Li N, Wang YHA, Iliev MN, Klein TM, Gupta A (2011). Growth of atomically smooth epitaxial nickel ferrite films by direct liquid injection CVD. Chem. Vap. Depos..

[24] Kahmei RR, Sai R, Arackal S, Shivashankar SA, Bhat N (2019). Nanostructured Zn-substituted nickel ferrite thin films: CMOS-compatible deposition and excellent soft magnetic properties. IEEE Magn. Lett..

[25] Jaffari GH, Rumaiz AK, Woicik JC, Shah SI (2012). Influence of oxygen vacancies on the electronic structure and magnetic properties of NiFe2O4 thin films. J. Appl. Phys..

[26] Emori S, Gray BA, Jeon HM, Peoples J, Schmitt M, Mahalingam K, Hill M, McConney ME, Gray MT, Alaan US, Bornstein AC (2017). Coexistence of low damping and strong magnetoelastic coupling in epitaxial spinel ferrite thin films. Adv. Mater..

[27] Singh AV, Khodadadi B, Mohammadi JB, Keshavarz S, Mewes T, Negi DS, Datta R, Galazka Z, Uecker R, Gupta A (2017). Bulk single crystal-like structural and magnetic characteristics of epitaxial spinel ferrite thin films with elimination of antiphase boundaries. Adv. Mater..

[28] Zhou P, Singh AV, Li Z, Popov MA, Liu Y, Filippov DA, Zhang T, Zhang W, Shah PJ, Howe BM, McConney ME (2019). Magnetoelectric interactions in composites of ferrite films on lattice-matched substrates and ferroelectrics. Phys. Rev. Appl..

[29] Regmi S, Li Z, Srivastava A, Mahat R, Kc S, Rastogi A, Galazka Z, Datta R, Mewes T, Gupta A (2021). Structural and magnetic properties of NiFe2O4 thin films grown on isostructural lattice-matched substrates. Appl. Phys. Lett..

[30] Robertson JM, Jansen M, Hoekstra B, Bongers PF (1977). Growth of spinel ferrite films by liquid phase epitaxy. J. Cryst. Growth.

[31] Zhou P, Popov MA, Liu Y, Bidthanapally R, Filippov DA, Zhang T, Qi Y, Shah PJ, Howe BM, McConney ME, Luo Y (2019). Converse magnetoelectric effects in composites of liquid phase epitaxy grown nickel zinc ferrite films and lead zirconate titanate: Studies on the influence of ferrite film parameters. Phys. Rev. Mater..

[32] Van der Straten PJM, Metselaar R (1980). Liquid phase epitaxial growth of lithium ferrite-aluminate films. J. Cryst. Growth.

[33] Van der Straten PJM, Metselaar R (1980). LPE growth of Mn, Ni-and Al-substituted copper ferrite films. J. Appl. Phys..

[34] van der Straten PJM, Metselaar R (1978). Stress-induced anisotropy in LPE grown Ni (Fe, Al) 2O4 films. Mater. Res. Bull..

[35] Krysztofik A, Özoğlu S, McMichael RD, Coy E (2021). Effect of strain-induced anisotropy on magnetization dynamics in Y_3_Fe_5_O_12_ films recrystallized on a lattice-mismatched substrate. Sci. Rep..

[36] Wynne R, Daneu JL, Fan TY (1999). Thermal coefficients of the expansion and refractive index in YAG. Appl. Opt..

[37] Mee JE, Archer JL, Meade RH, Hamilton TN (1967). Chemical vapor deposition of epitaxial YIG on YAG and epitaxial GdIG on YAG. Appl. Phys. Lett..

[38] Li N, Wang Y-H, Iliev MN, Klein TM, Gupta A (2011). Growth of atomically smooth epitaxial nickel ferrite films by direct liquid injection CVD. Chem. Vap. Deposition.

[39] Althammer M, Singh AV, Wimmer T, Galazka Z, Huebl H, Opel M, Gross R, Gupta A (2019). Role of interface quality for the spin Hall magnetoresistance in nickel ferrite thin films with bulk-like magnetic properties. Appl. Phys. Lett..

[40] Smith AB, Jones RV (1966). Magnetostriction in nickel ferrite and cobalt—Nickel ferrite. J. Appl. Phys..

[41] Bozorth RM, Walker JG (1952). Magnetostriction of single crystals of cobalt and nickel ferrites. Phys. Rev..

[42] Kittel C (1958). Excitation of spin waves in a ferromagnet by a uniform rf field. Phys. Rev..

[43] Bursian VE, Kaveev AK, Korovin AM, Krichevtsov BB, Lutsev LV, Suturin SM, Sawada M, Sokolov NS (2019). Bulk-Like Dynamic Magnetic Properties of Nickel Ferrite Epitaxial Thin Films Grown on SrTiO_3_ (001) Substrates. IEEE Magn. Lett..

[44] Liu, Y., Mei, Z., Guo, Y., Zhou, P., Qi, Y., Liang, K., Ma, Z., Xia, Z., Adhikary, A., Dong, C. & Sun, N. Evidence for strain control of magnetic anisotropy in epitaxial nickel ferrite thin films grown on strontium titanate substrates. *Mater. Res. Bull. ***138,** 111214 (2021).

