# Supplementary material for: Strain induced anisotropy in liquid phase epitaxy grown nickel ferrite on magnesium gallate substrates
Source: Sci Rep. 2022 Apr 29;12:7052. doi: 10.1038/s41598-022-10814-8 (PMC9054777; doi:10.1038/s41598-022-10814-8)
Supplement: Supplementary file 1 — Supplementary Information. [file 41598_2022_10814_MOESM1_ESM.pdf]

SUPPLEMENT TO:

Strain Induced Anisotropy in Liquid Phase Epitaxy Grown Nickel Ferrite on Magnesium Gallate Substrates

Ying Liu, [1,2] Peng Zhou,[2] Sudhir Regmi,[3] Rao Bidthanapally,[1] Maksym Popov,[4] Jitao Zhang,[5] Wei Zhang,[1] M.R. Page,[6] T. Zhang, [2] Arunava Gupta,[3] and G. Srinivasan[1]

[1] *Department of Physics, Oakland University, Rochester, MI 48309, USA*

[2] *Department of Materials Science and Engineering, Hubei University, Wuhan 430062, China*

[3] *Center for Materials for Information Technology, The University of Alabama, Tuscaloosa, AL 3548, USA*

[4] *Faculty of Radiophysics, Electronics and Computer Systems, Taras Shevchenko National University of Kyiv, Kyiv, 01601, Ukraine*

[5] *College of Electrical and Information Engineering, Zhengzhou University of Light Industry, Zhengzhou 450002, China*

[6] *Materials and Manufacturing Directorate, Air Force Research Laboratory, Wright-Patterson Air Force Base, Dayton, Ohio 45433, USA*

Corresponding author: G. Srinivasan
email: srinivas@oakland.edu

2.5 µm thick NFO film on (100) MGO

SEM Image at film-substrate cross-section and EDX of sample surface:

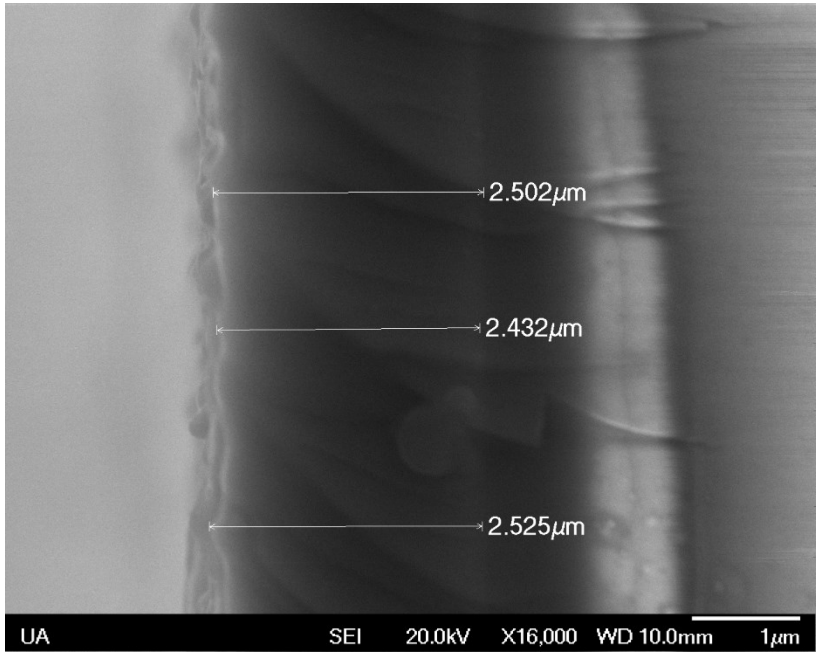

cross-sectional SEM for sample thickness

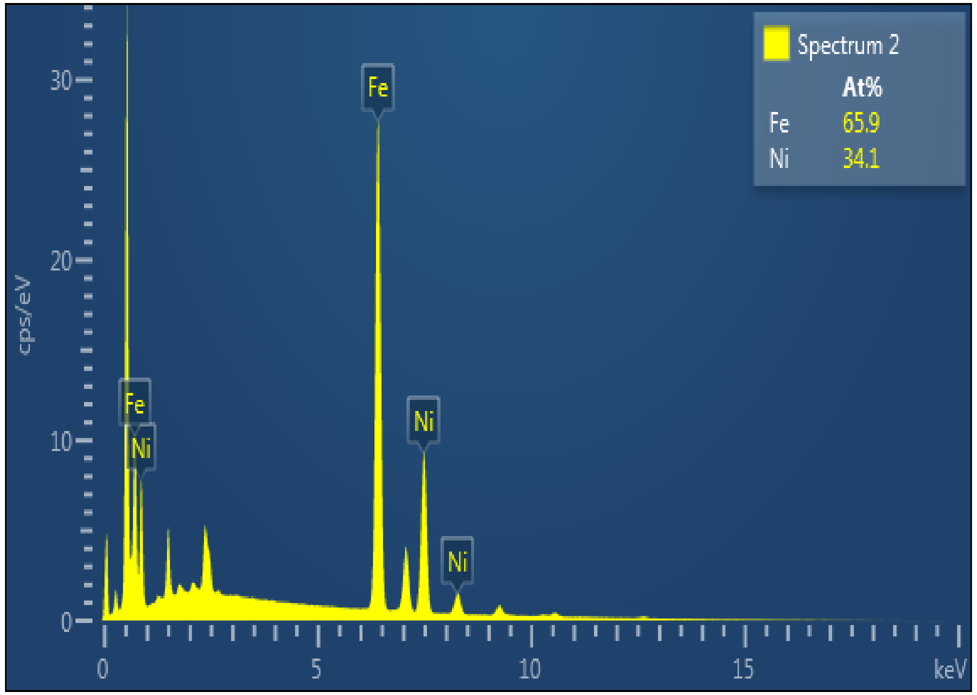

Figure S1: Cross-sectional SEM and energy dispersive X-ray diffracton (EDX) data for 2.5 micron thick nickel ferrite (NFO) film on (100) magnesium gallate (MGO) substrate.

10 μm thick NFO film on (100) MGO

SEM Image for thickness measurement

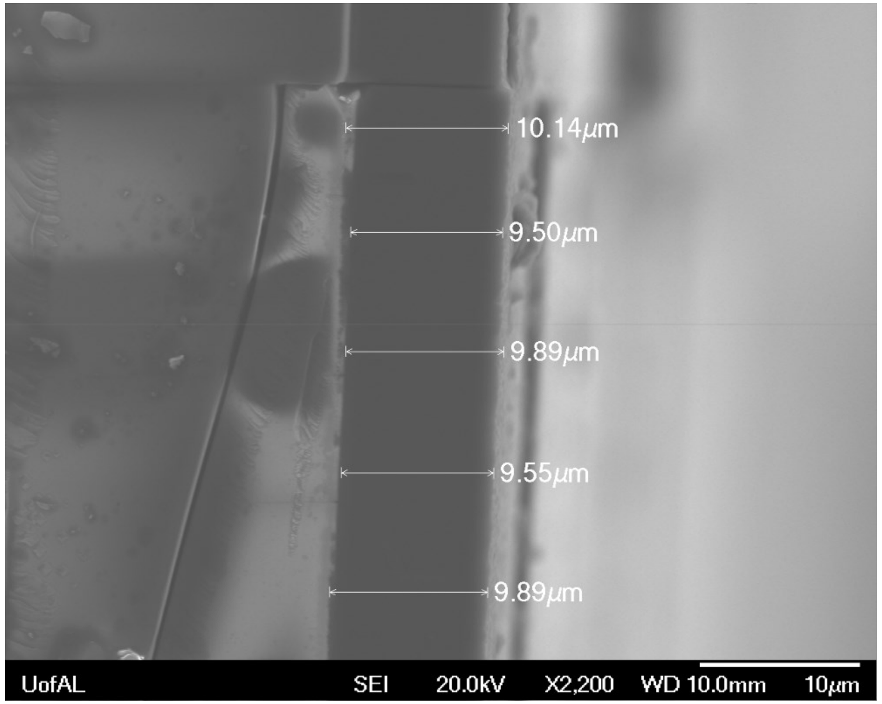

Figure S2: Cross-sectional SEM image for 10 micron thick NFO film on (100) MGOsubstrate.

7.5 µm thick NFO film on (110) MGO

SEM at sample cross-section and EDX of sample surface:

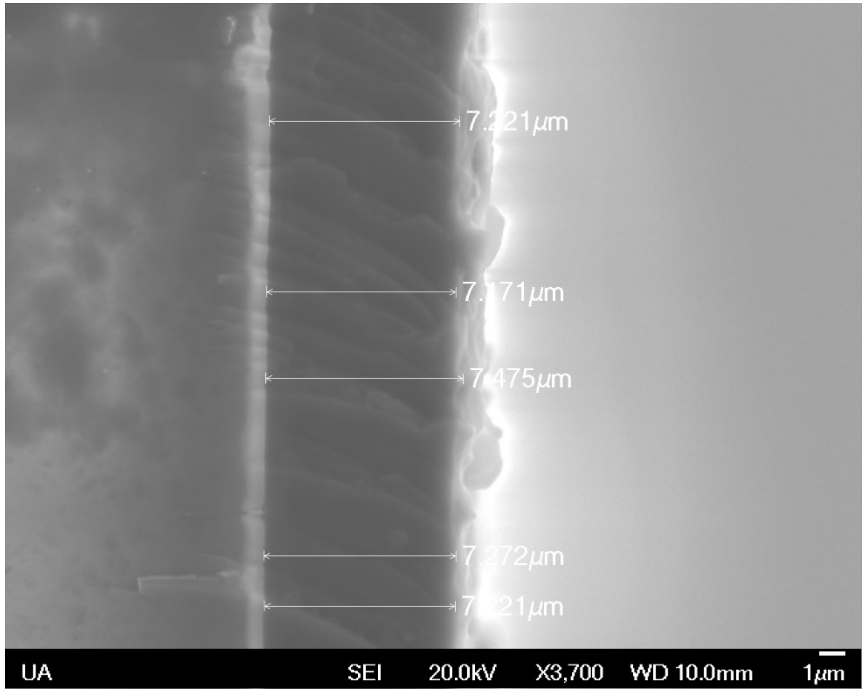

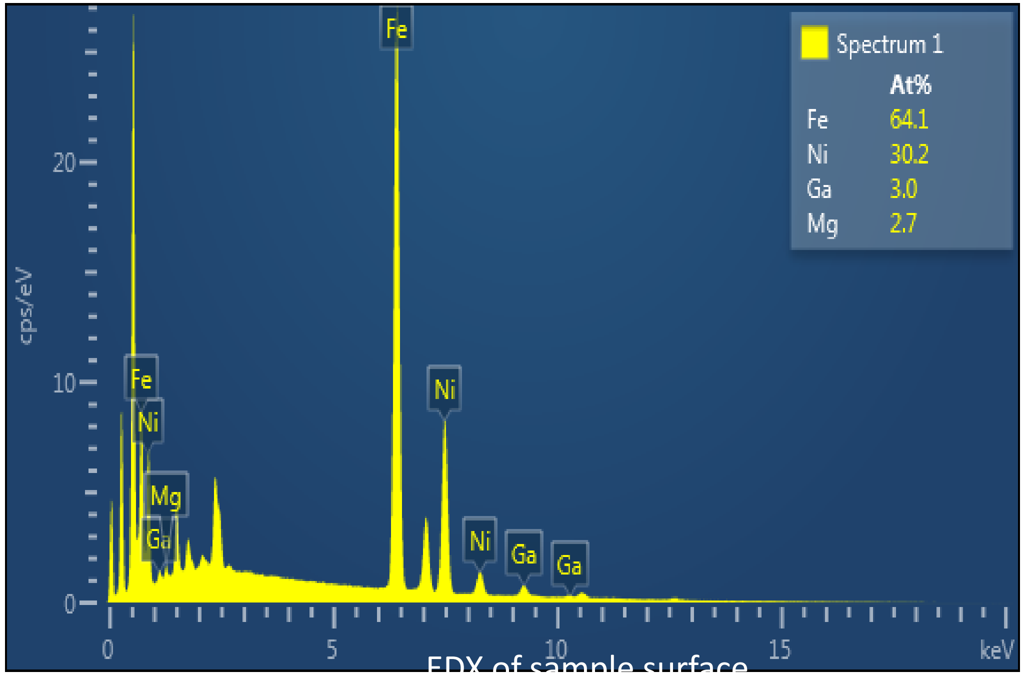

Figure S3: Cross-sectional SEM and energy dispersive X-ray diffracton (EDX) data for 7.5 micron thick nickel ferrite (NFO) film on (110) magnesium gallate (MGO) substrate.

10 μm thick NFO film on (110) MGO

<u>SEM Image at sample cross-section and EDX of sample surface</u>:

cross-sectional SEM for sample thickness

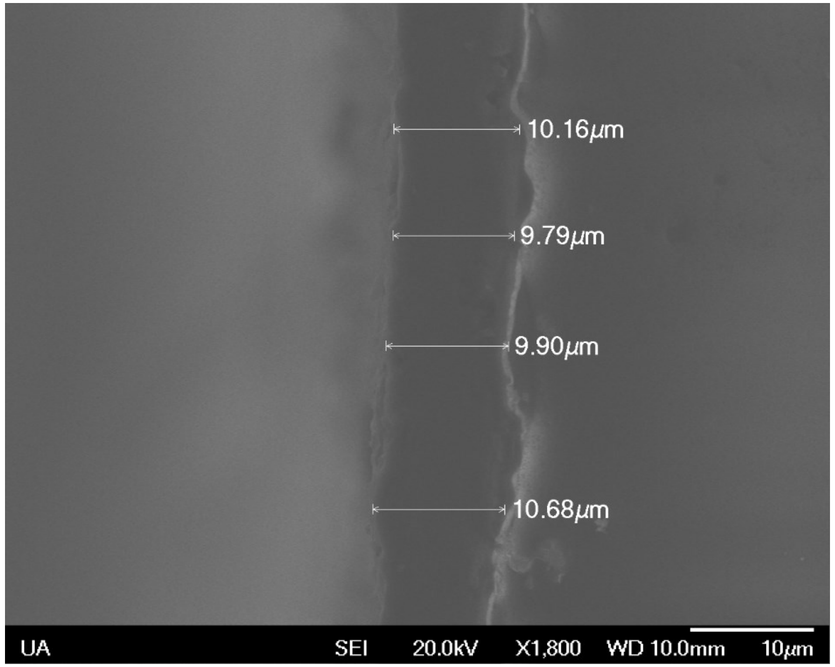

EDX of sample surface

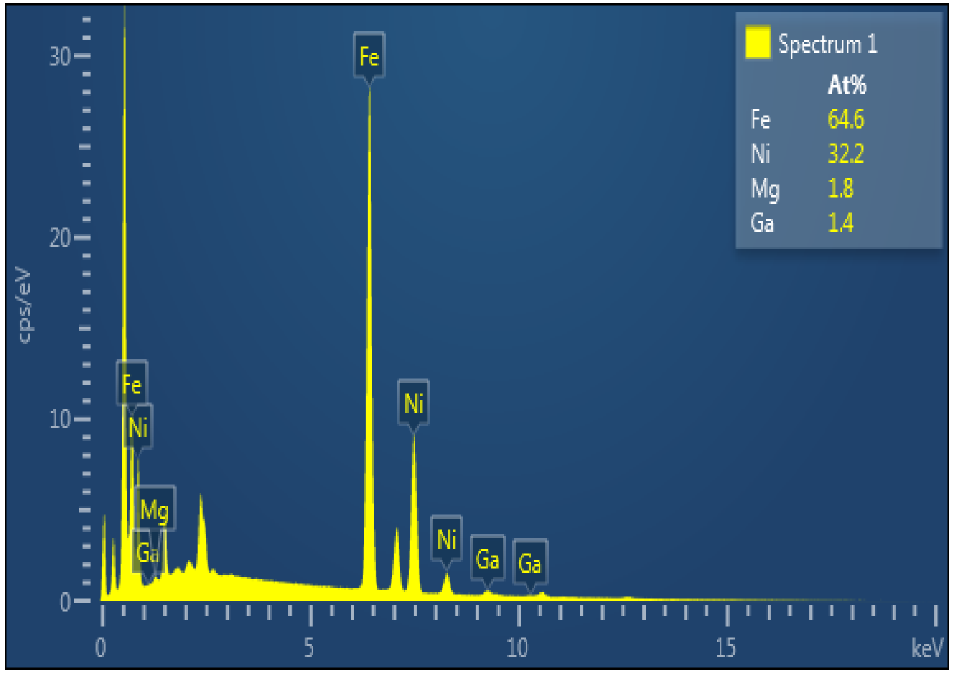

Figure S4: Cross-sectional SEM and energy dispersive X-ray diffracton (EDX) data for 10 micron thick nickel ferrite (NFO) film on (110) magnesium gallate (MGO) substrate.

5 μm thick NFO film on (110) MGO

Surface Topography Image using an Atomic Force microscope

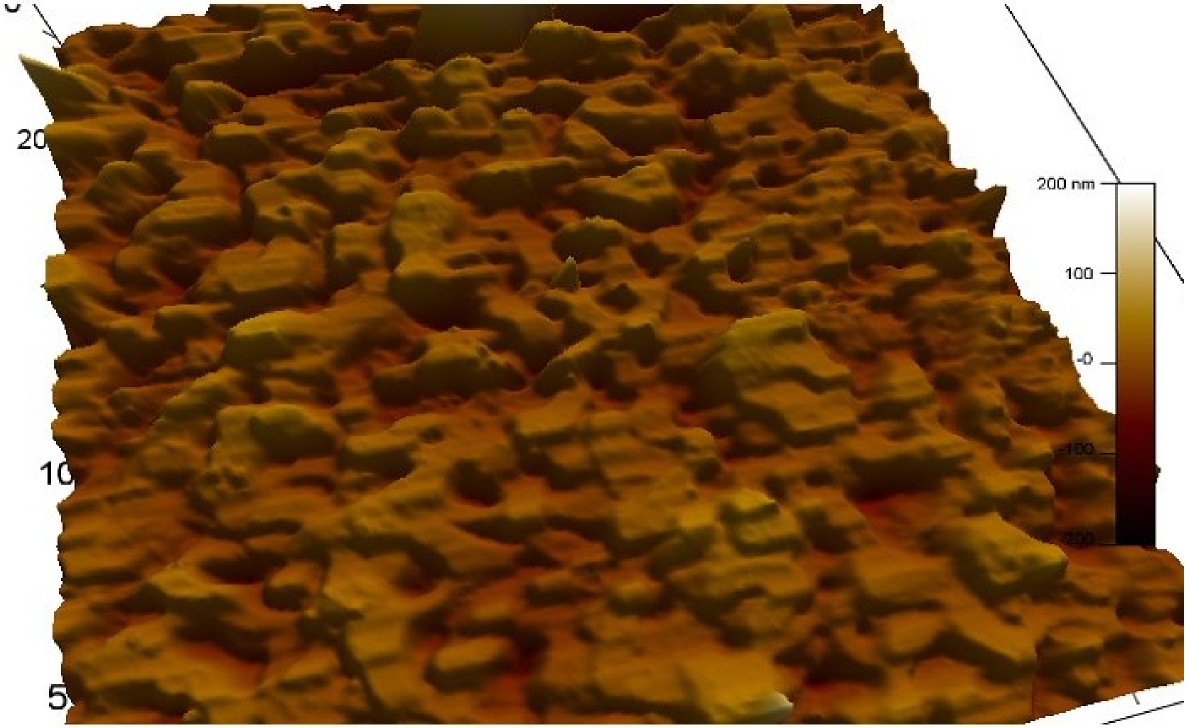

AFM Image for surface roughness measurement

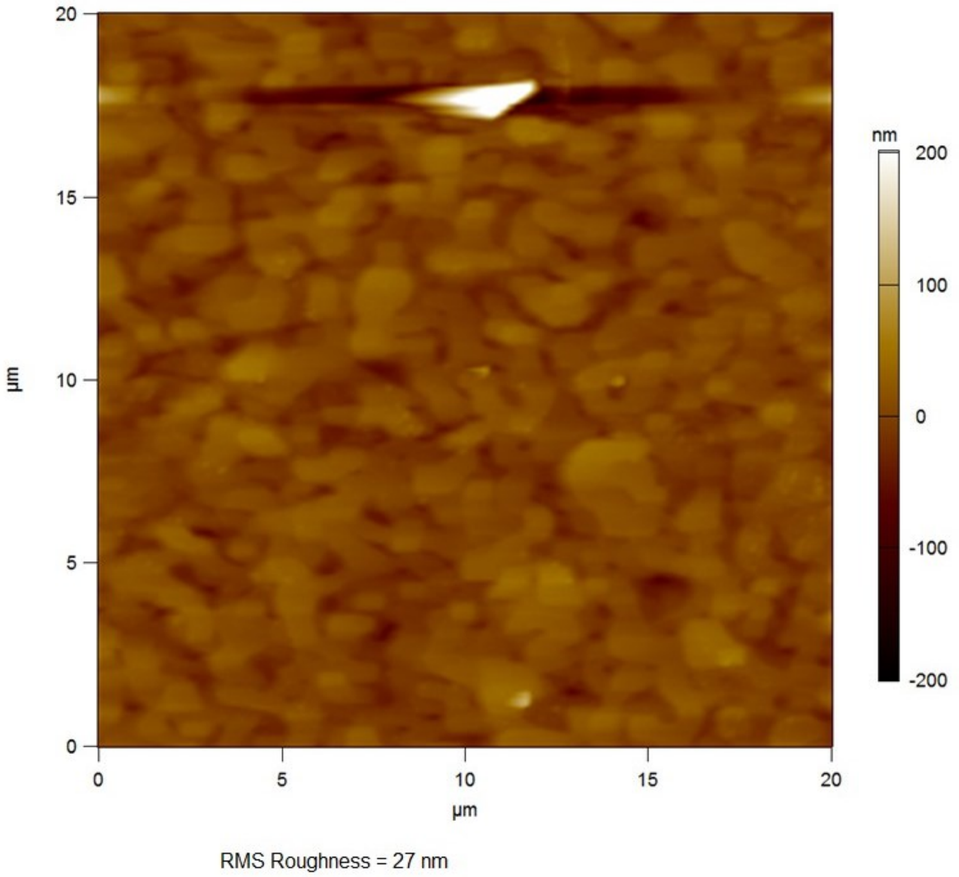

Figure S5: (Left) AFM topography images of NFO film of thickness of 5 μm on (110) MGO substrate. (Right) AFM Image surface roughness. The RMS roughness is 27 nm.

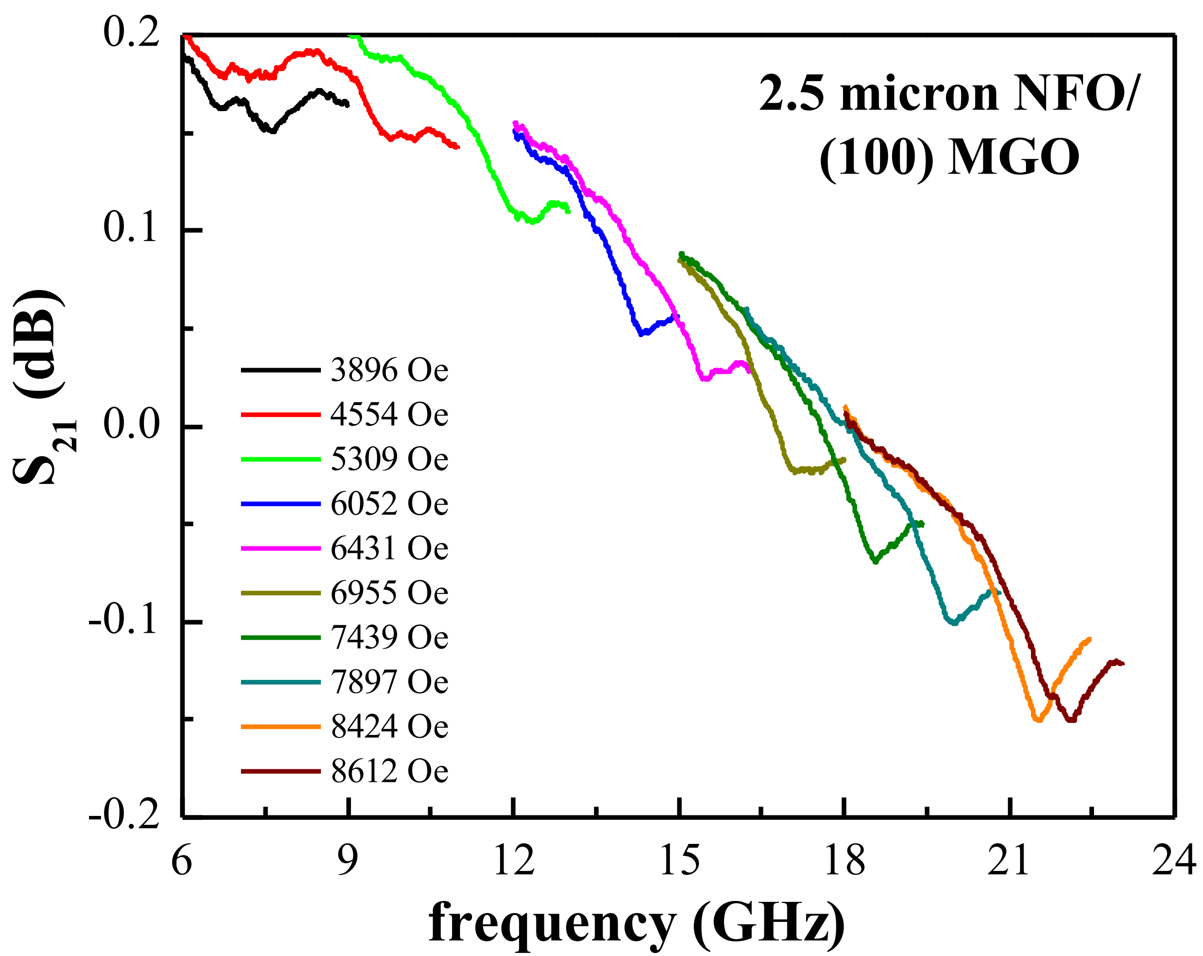

Figure S6: Profiles of scattering matrix S21 vs H showing FMR in 2.5 micron thick NFO film on (100) MGO for a series of static fields applied perpendicular to the sample plane.

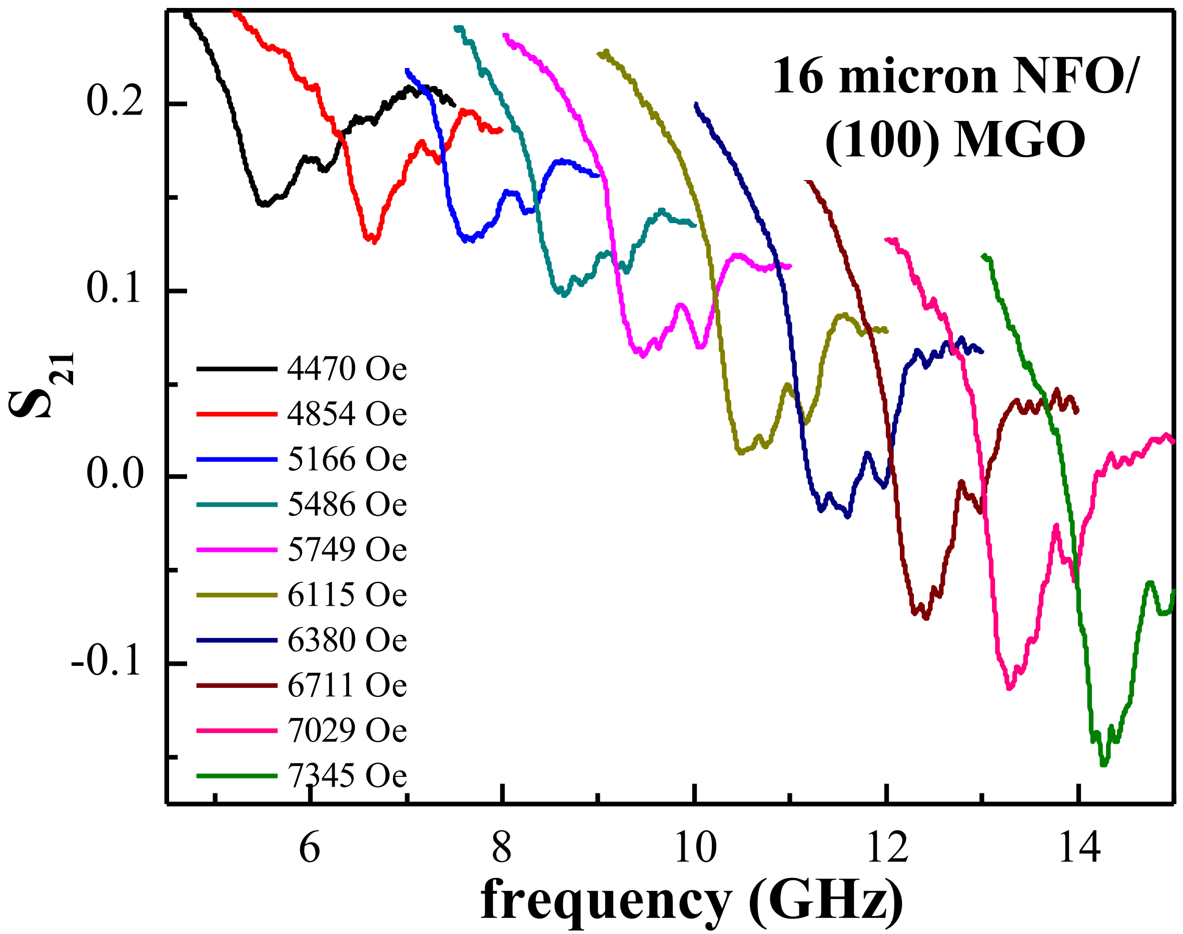

Figure S7: Profiles of scattering matrix S_{21} vs H showing FMR in 16 micron thick NFO film on (100) MGO for a series of static fields applied perpendicular to the sample plane.

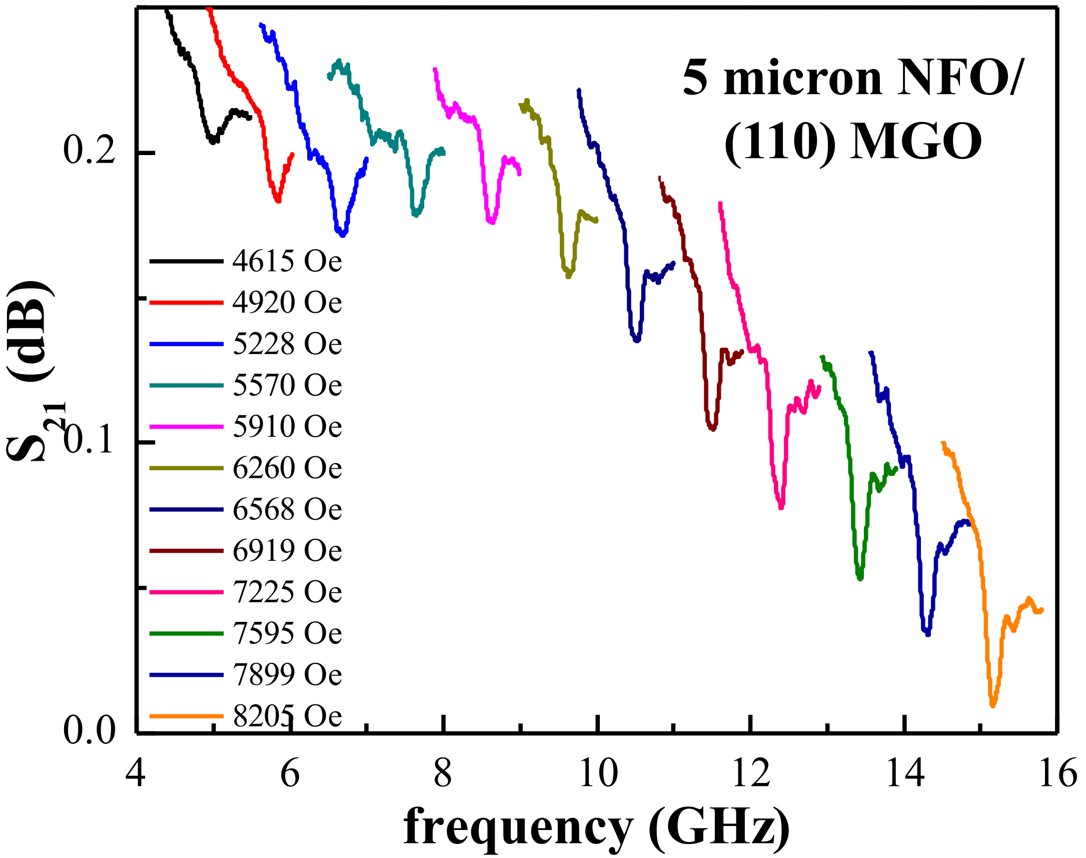

Figure S8: Profiles of scattering matrix S_{21} vs H showing FMR in 5 micron thick NFO film on (110) MGO for a series of static fields applied perpendicular to the sample plane.

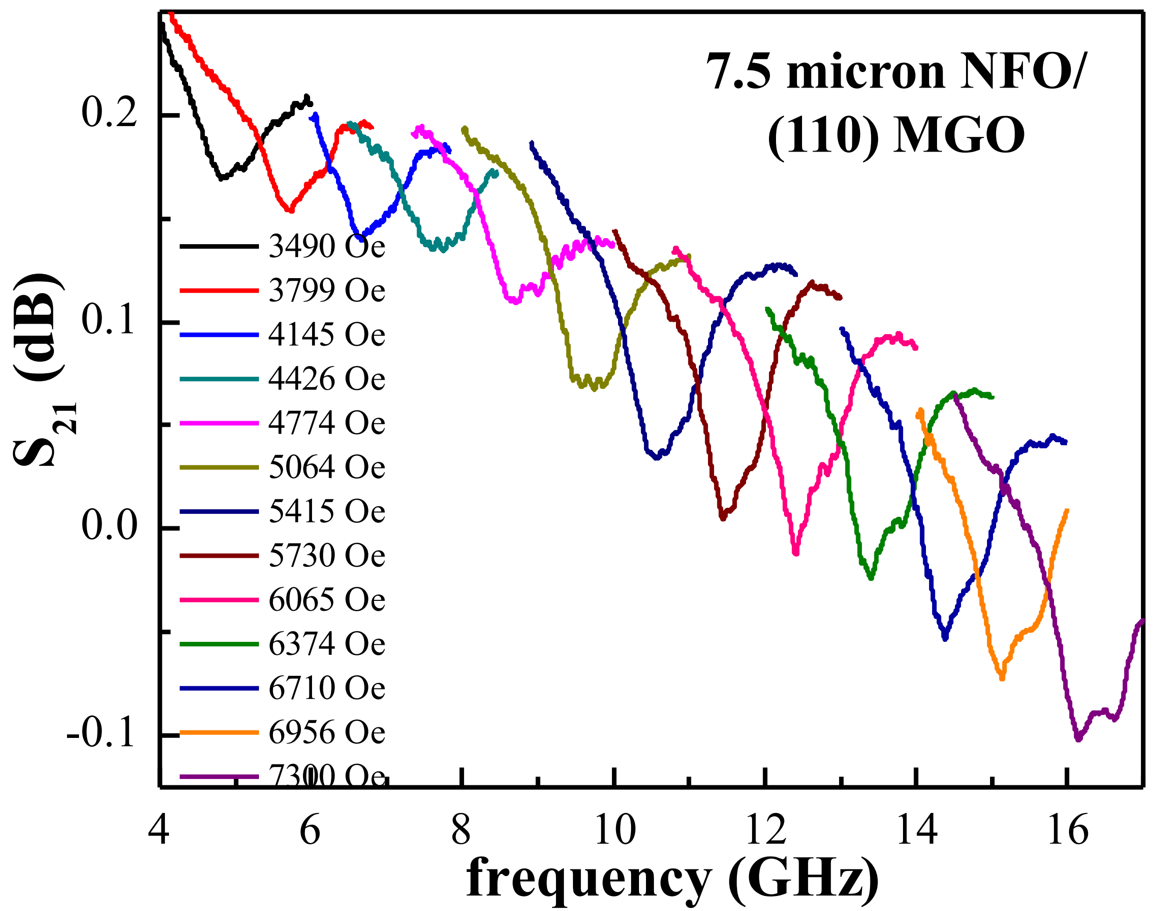

Figure S9: Profiles of scattering matrix S21 vs H showing FMR in 7.5 micron thick NFO film on (110) MGO for a series of static fields applied perpendicular to the sample plane.

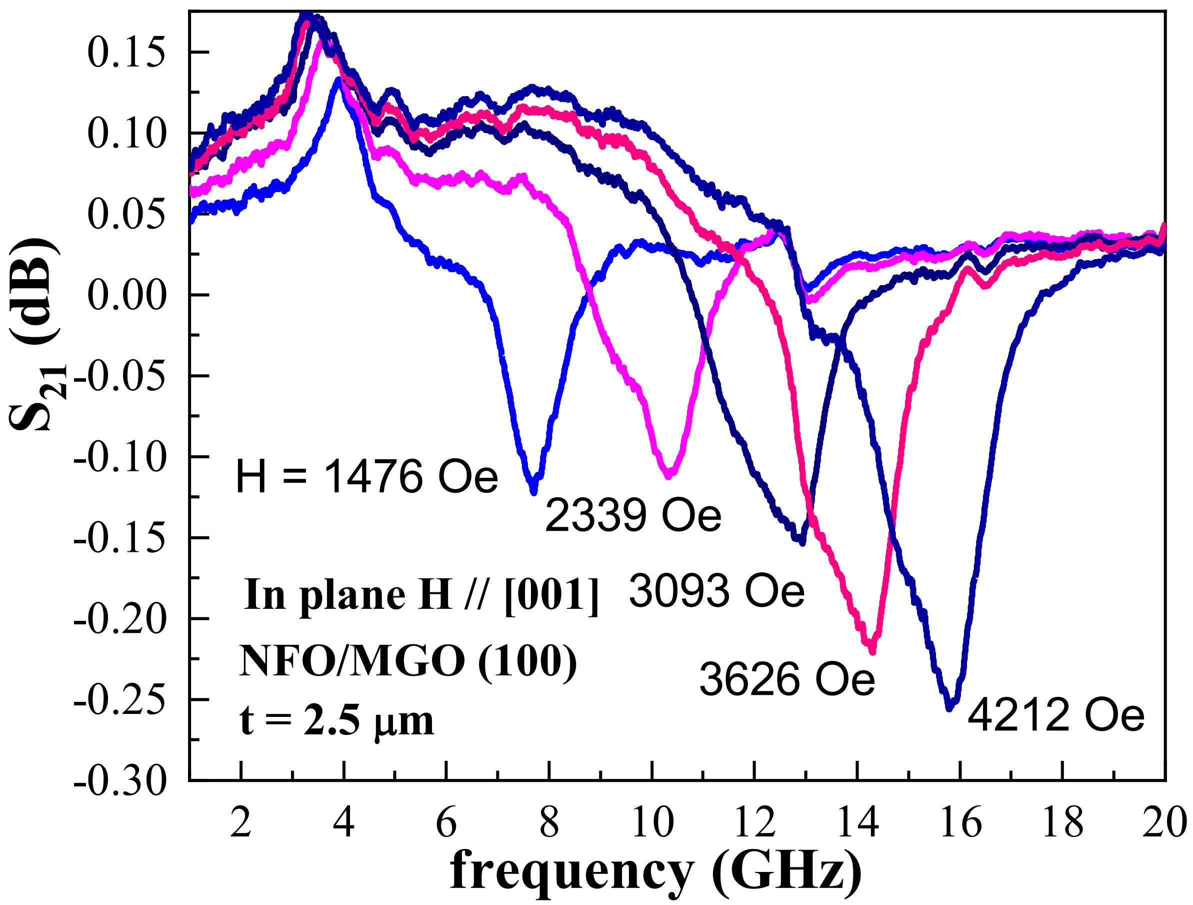

Figure S10: Profiles of S21 vs f showing FMR in 2.5 micron thick NFO film on (100) MGO for H // [001].

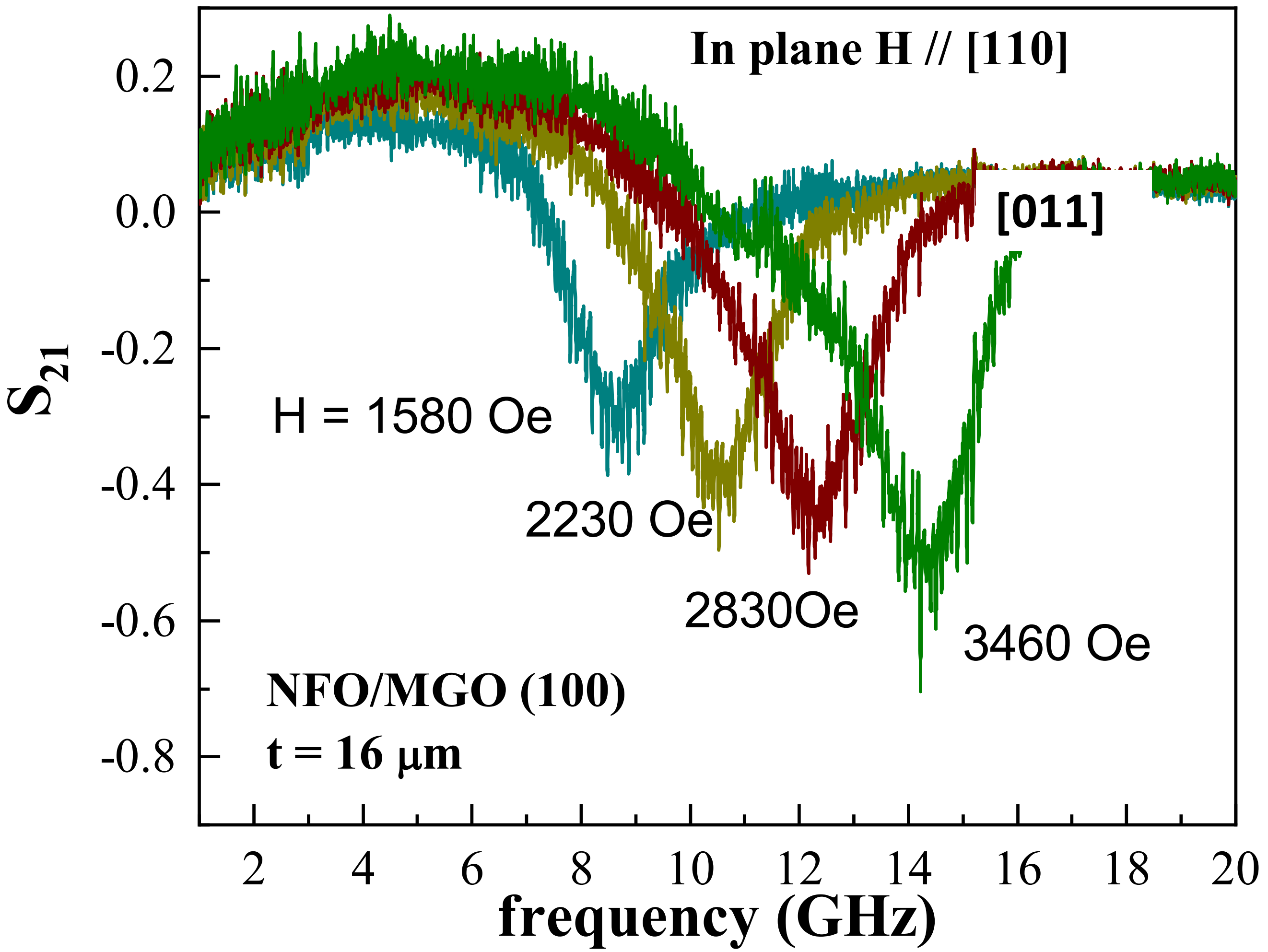

Figure S11: Profiles of S21 vs f showing FMR in 16 micron thick NFO film on (100) MGO for H // [001].

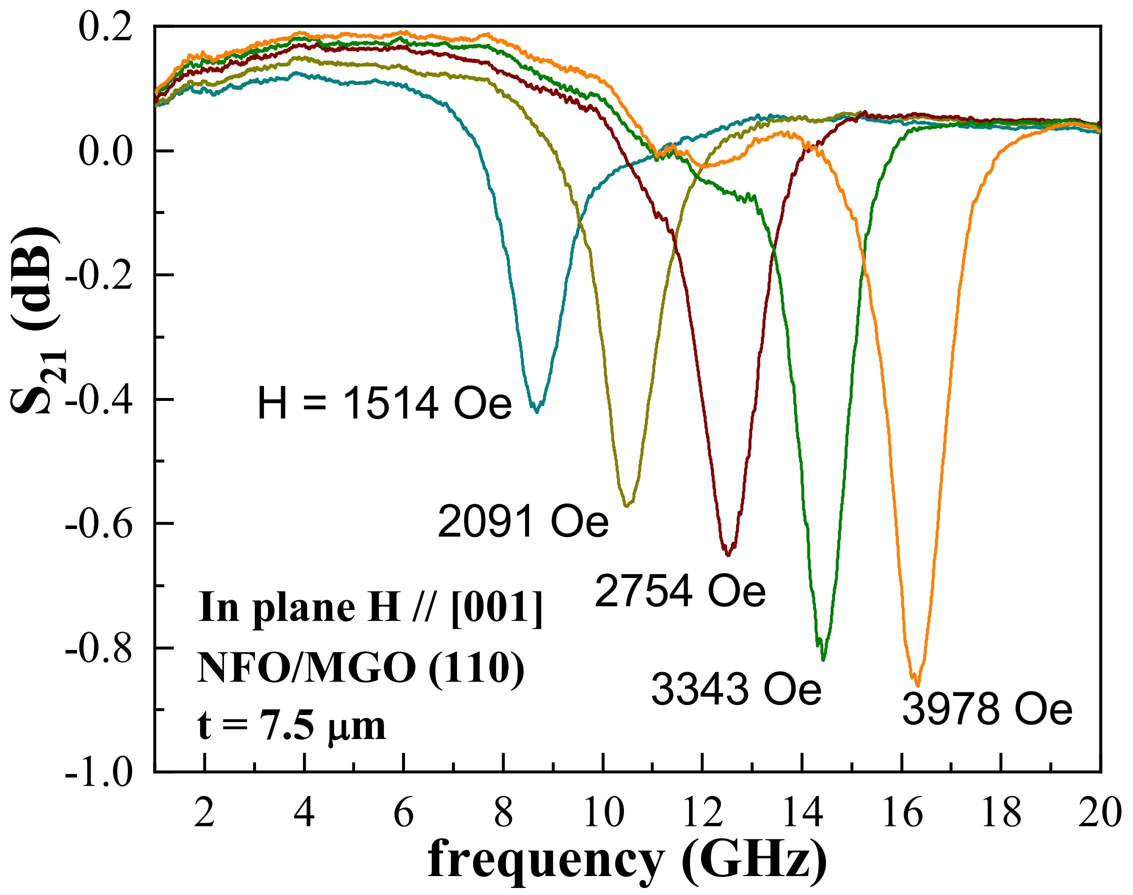

Figure S12: Profiles of S21 vs f showing FMR in 7.5 micron thick NFO film on (110) MGO for H // [001].

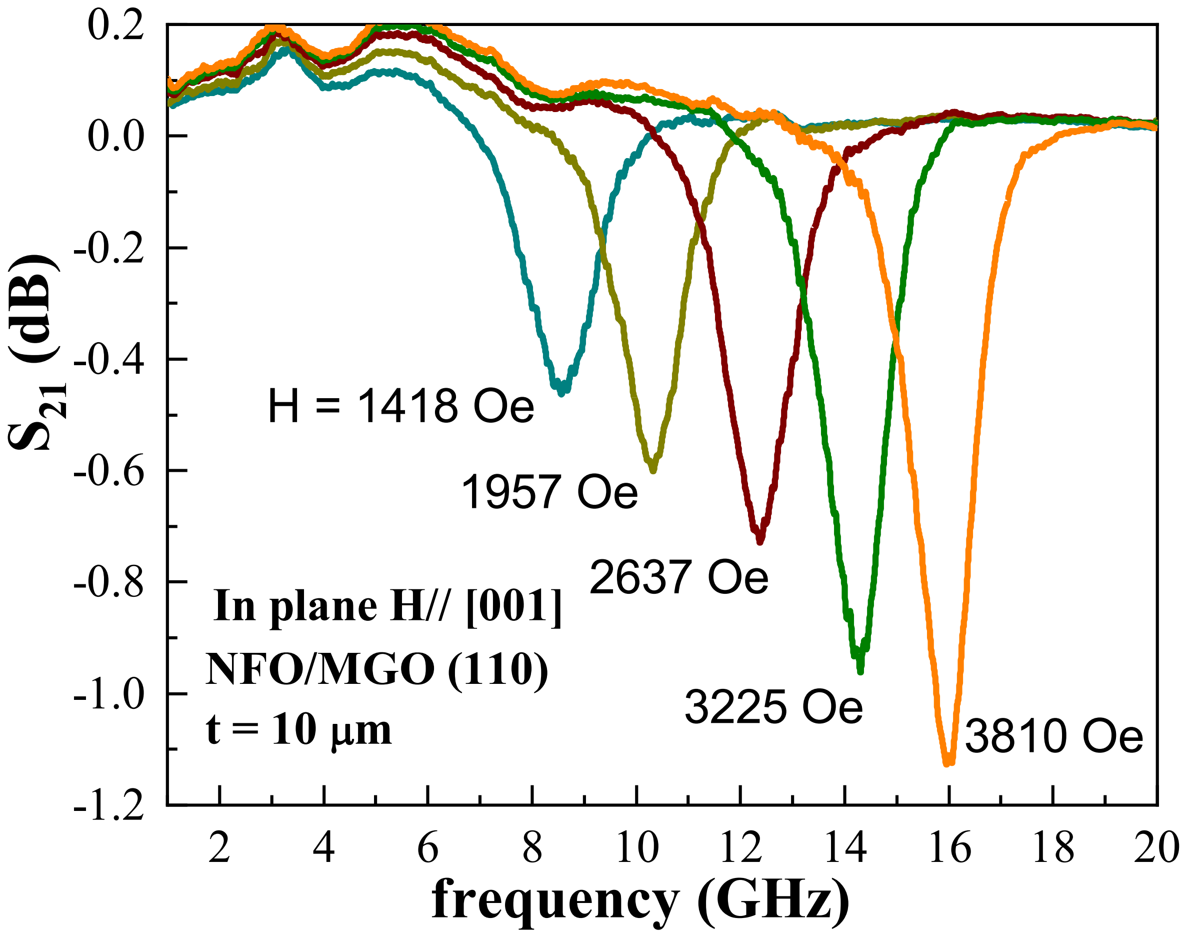

Figure S13: Profiles of S21 vs f showing FMR in 10 micron thick NFO film on (110) MGO for H // [001].
